# Vicenin-2 attenuates rosacea-like inflammation by inhibiting IL-17RA signaling

**DOI:** 10.3389/fphar.2026.1793115

**Published:** 2026-03-10

**Authors:** Meng Chen, Xinyi Peng, Wenlong Shuai, Jia He, Hao Yang, Bowen Tan, Yan Zhou, Lei Zhang, Xi Duan

**Affiliations:** Department of Dermatovenereology, Affiliated Hospital of North Sichuan Medical College, Nanchong, Sichuan, China

**Keywords:** ACT1, IL-17RA/NF-κB, p65, rosacea, TRAF6, Vicenin-2

## Abstract

**Introduction:**

Rosacea is a persistent inflammatory dermatosis with a multifactorial etiology that significantly impairs patients’ daily life and wellbeing. Vicenin-2, a natural flavonoid, has shown anti-inflammatory potential; however, its therapeutic mechanism in rosacea remains unclear. We seek to understand the potential and mechanism of Vicenin-2 in the treatment of rosacea.

**Methods:**

Differentially expressed genes (DEGs) related to rosacea were identified from the Gene Expression Omnibus (GEO) dataset GSE65914 using GEO2R. Potential targets of Vicenin-2 were predicted via PharmMapper and SwissTargetPrediction, and overlapping targets were selected. Gene Ontology (GO) and Kyoto Encyclopedia of Genes and Genomes (KEGG) pathway enrichment analyses were conducted using DAVID. Core targets were identified through STRING and Cytoscape, and molecular docking was performed to evaluate binding affinity. *In vivo* rosacea mouse models and *in vitro* HaCaT cell models were established to validate therapeutic effects using histological staining, immunofluorescence, immunohistochemistry, Enzyme-Linked Immunosorbent Assay (ELISA), Reverse Transcription Quantitative Polymerase Chain Reaction (RT-qPCR), and Western blotting.

**Results:**

Forty-three candidate targets of Vicenin-2 were identified. Enrichment analyses indicated that these targets were mainly involved in immune regulation and inflammatory responses, with the IL-17 signaling pathway as a key pathway. Molecular docking revealed strong binding between Vicenin-2 and MMP1 and MMP3. Experimental results showed that topical Vicenin-2 significantly reduced erythema and inflammatory cell infiltration in mice. Both *in vivo* and *in vitro* assays confirmed that Vicenin-2 modulates the IL-17RA/NF-κB pathway, suppressing the expression of inflammatory mediators and matrix metalloproteinases.

**Discussion:**

Vicenin-2 alleviates LL-37-induced rosacea-like inflammation by regulating the IL-17RA/NF-κB pathway and exerting multi-target anti-inflammatory and immunomodulatory effects, suggesting its potential as a novel therapeutic agent for rosacea. Trial registration: not applicable.

## 1 Background

Rosacea is a chronic, recurrent inflammatory skin condition that predominantly affects young to middle-aged women (Clanner-Engelshofen et al., 2022). Its typical clinical manifestations include facial flushing and telangiectasia in the central face, frequently associated with burning sensation and pruritus (Crawford et al., 2004; Thiboutot et al., 2020). Updated research indicates that rosacea affects approximately 5.46% of adults (Gether et al., 2018), especially prevalent in individuals with light skin (Fitzpatrick types I–II) and those of Northern European descent (Rainer et al., 2017; Sarkar et al., 2020). Its pathogenesis is complex, involving multiple factors, including genetic susceptibility, immune dysregulation, neurovascular dysfunction, and compromised skin barrier integrity (Grafanaki et al., 2025). Dysregulated activation of the innate immune system is considered a central mechanism (Del et al., 2012). LL-37, an antimicrobial peptide synthesized by keratinocytes and other sources, is abnormally overexpressed in rosacea. It initiates and amplifies downstream inflammatory cascades by activating pathways such as Toll-like receptor 2 (TLR2) (Jiang et al., 2020; Yamasaki et al., 2007). However, the specific mechanism still requires further investigation.

Standard rosacea treatments include topical ivermectin, metronidazole, azelaic acid, vasoconstrictors, and oral doxycycline, but relapses and significant side effects persist. Recently, natural plant compounds, known for their low toxicity and stable efficacy, have been increasingly used for the treatment of rosacea. Compounds like Oroxylin A (OA) and Epigallocatechin-3-gallate (EGCG) have shown potential to slow immune changes, inflammation, and angiogenesis by downregulating the SIRT3-SOD2 axis and inducing autophagy, demonstrating strong potential for application in rosacea (Feng et al., 2024; Meng et al., 2024; Zhou et al., 2023). Vicenin-2 is a flavonoid found in medicinal plants. It exhibits diverse pharmacological activities, including anti-inflammatory, immunomodulatory, and anti-angiogenic effects (Kang et al., 2015; Marrassini et al., 2011). Our previous findings demonstrated that Vicenin-2 has strong abilities to fight free radicals, reduce inflammation, and prevent cell death in skin photoaging (Duan et al., 2019; Hu et al., 2025). Critically, skin photoaging and rosacea exhibit significant overlap in etiological factors and pathogenesis, including exposure to ultraviolet (UV) radiation, chronic inflammation, immune activation, and the upregulation of matrix metalloproteinases (MMPs), which are very important in the development of both conditions (Shin et al., 2025; Guo et al., 2025; Zhu et al., 2025; Yang et al., 2024). Consequently, we speculate that Vicenin-2 might also help with rosacea; however, the fundamental molecular mechanisms remain to be fully understood.

Integrating molecular docking, network pharmacology, and bioinformatics offers powerful tools for understanding the multi-target, multi-pathway mechanisms of natural products. By analyzing disease transcriptomics, key genes and pathways are identified, while network pharmacology builds “drug-target-disease” networks to predict mechanisms. Molecular docking confirms these predictions by assessing binding mechanisms and affinities between drugs and target proteins (Wang et al., 2026; Zhao et al., 2023). This study used Gene expression profiles from the Gene Expression Omnibus (GEO) database to screen rosacea-related targets with GEO2R. Network pharmacology identified potential Vicenin-2 targets. Gene Ontology (GO) and Kyoto Encyclopedia of Genes and Genomes (KEGG) analyses revealed its functional roles and pathways. Molecular docking validated the binding potential. Rosacea mouse and HaCaT cell models were used to evaluate Vicenin-2’s therapeutic efficacy and mechanisms. This work provides a theoretical and experimental basis for Vicenin-2 as a potential rosacea treatment and offers insights into related natural products.

## 2 Materials and methods

### 2.1 Acquisition of rosacea targets

Rosacea-related gene expression datasets were retrieved from the GEO database using “rosacea” as the search term. Among the identified human transcriptome datasets, GSE65914 (GPL570 platform) was selected for further analysis. Differentially expressed genes (DEGs) among rosacea subtypes, including erythematotelangiectatic rosacea (ETR), papulopustular rosacea (PPR), and phymatous rosacea (PHR), were identified using GEO2R. Genes with a P-value <0.05 and |logFC| ≥ 1 were defined as DEGs. Overlapping DEGs across different subtypes were determined using Venny 2.1 for subsequent analyses.

### 2.2 Identification of Vicenin-2 targets and prediction of therapeutic targets of Vicenin-2 for rosacea

Vicenin-2 targets were predicted using its SMILES from the PubChem database. Potential therapeutic targets for rosacea were identified through three databases: SwissTargetPrediction, PharmMapper, and SEA. The overlapping targets from these databases and Section 2.1 were identified using Venny 2.1 as potential therapeutic targets of Vicenin-2 for rosacea.

### 2.3 GO and KEGG enrichment analyses of Vicenin-2-rosacea targets

The potential targets identified in Section 2.2 were subjected to Gene Ontology and KEGG pathway enrichment analyses using the DAVID database, with p < 0.05 as the cutoff. Enrichment results were visualized using the Bioinformatics platform.

### 2.4 Construction of the PPI network and identification of key genes

A protein–protein interaction (PPI) network was constructed using the STRING database and analyzed with Cytoscape (v3.7.1). Hub genes were identified using the CytoHubba plugin based on the maximal clique centrality (MCC) algorithm, and the top 10 hub targets were selected for further analysis.

### 2.5 Molecular docking and visualization

The 3D structure of Vicenin-2 was obtained from PubChem and converted to MOL2 format using Chem3D, while target protein structures were retrieved from the RCSB PDB. Proteins were prepared by removing ligands and water molecules using PyMOL, followed by hydrogen addition, charge calculation, and atom type assignment with AutoDock Tools (v1.5.6), and saved in PDBQT format. Molecular docking was performed using AutoDock Vina (v1.1.2), and docking results were visualized and analyzed with PyMOL.

### 2.6 Animal experiments

Thirty female BALB/c mice (20–22 g, 6–8 weeks) were obtained from Chengdu Bio-Rey Biotechnology Company. LL-37 (1 mg; GLPBIO, GC14377, >98% purity) was dissolved in 348 μL PBS to prepare a 640 μM solution, and Vicenin-2 (1 mg; GLPBIO, GC37902, >98% purity) was dissolved in 8.42 μL 0.1% DMSO and diluted with PBS to the desired concentrations. All solutions were freshly prepared. Mice were randomly divided into six groups (n = 5): control, LL-37, LL-37 + vehicle, and LL-37 combined with Vicenin-2 at 0.025%, 0.05%, or 0.1% (w/v). Except for the control and LL-37 groups, mice received topical application of Vicenin-2 or vehicle for 7 consecutive days. Except for the control group mice, on the 6th and 7th days, after inhalation anesthesia with 2% isoflurane, 20 μL of 640 μM LL - 37 was intradermally injected into the same area of the back (once every 12 h, a total of 4 injections) to induce rosacea-like dermatitis. On day 8, dorsal erythema was photographed and quantified using ImageJ, followed by severity scoring. Subsequently, the mice were euthanized by cervical dislocation after being anesthetized with an intraperitoneal injection of sodium pentobarbital at a dose of 100–200 mg/kg. The dorsal skin tissues were collected for further analysis. All procedures were approved by the Animal Ethics Committee of North Sichuan Medical College (NSMC2025-DW-011) and performed in accordance with the American Veterinary Medical Association (AVMA) standards for animal care and euthanasia.

### 2.7 Histological analysis

Mouse skin was fixed in 4% paraformaldehyde, dehydrated, embedded in paraffin, sectioned, and stained with HE and toluidine blue. For immunohistochemistry (IHC), antigen retrieval was performed in Tris-EDTA buffer, and sections were incubated with a p-p65 primary antibody, followed by a secondary antibody and DAB visualization. Immunofluorescence (IF) used a CD4 antibody, followed by FITC-secondary antibody, DAPI staining, and imaging under a fluorescence microscope. Inflammatory cell counts (via HE staining) and mast cell counts (via Toluidine Blue staining) were performed by two independent, blinded observers. For each experimental group, three samples were randomly selected, and three random fields per slide were quantified under ×400 magnification; raw data are provided in Supplementary Tables S3, S4. Similarly, for IHC and IF quantitative analysis, two independent blinded observers selected three samples per group. For each sample, three identical-sized fields were captured at ×400 magnification. The integrated optical density (IOD) of IF images was analyzed using Image-Pro Plus 6.0 software. Raw data are available in Supplementary Tables S5, S6.

### 2.8 Cell culture and treatment

HaCaT cells were cultured in DMEM with 10% FBS and 1% antibiotics at 37 °C. LL-37 and Vicenin-2 stock solutions were prepared and diluted for treatment (2–6 μM) in DMEM, with DMSO concentrations below 0.1% (Zhang et al., 2016). Previous toxicological studies have suggested that while DMSO can exhibit cytotoxicity even at low concentrations, a concentration of 0.1% (v/v) is generally considered the safety threshold for cellular assays (Galvao et al., 2014). Cells were treated with LL-37 and/or Vicenin-2 for experiments.

### 2.9 Cell viability assay

HaCaT cells were seeded in a 96-well plate, incubated overnight, and treated with CCK-8 reagent to measure absorbance at 450 nm. The study groups included: 1) control, 2) model (LL-37 10 μM), 3) low concentration Vicenin-2 (LL-37 + 2 μM Vicenin-2), 4) medium concentration Vicenin-2 (LL-37 + 4 μM Vicenin-2), and 5) high concentration Vicenin-2 (LL-37 + 6 μM Vicenin-2).

### 2.10 ELISA

Skin tissues and HaCaT cells were homogenized, and cytokines (IL-8, IL-1β, IL-6, TNF-α) were measured using ELISA kits. CXCL1/KC was used as a functional IL-8 equivalent for rodents (Levin et al., 2014).

### 2.11 Real-time quantitative PCR

Total RNA was extracted from cells and tissues, reverse-transcribed, and quantified by SYBR Green-based qPCR. Transcript levels were normalized to GAPDH and quantified by the 2^−ΔΔCt^ method. Primer sequences are provided in Table 1.

**TABLE 1:** Summary table of primer sequences for animal and cell RT-qPCR experiments.

Name	Primer	Sequence
Mouse GAPDH	Forward	ATG​GGT​GTG​AAC​CAC​GAG​A
	Reverse	CAG​GGA​TGA​TGT​TCT​GGG​CA
Mouse TNF-α	Forward	CGT​CAG​CCG​ATT​TGC​TAT​CT
	Reverse	CGG​ACT​CCG​CAA​AGT​CTA​AG
Mouse IL-1β	Forward	TCA​GGC​AGG​CAG​TAT​CAC​TC
	Reverse	AGC​TCA​TAT​GGG​TCC​GAC​AG
Mouse IL-6	Forward	CTT​CCA​TCC​AGT​TGC​CTT​CTT
	Reverse	AAT​TAA​GCC​TCC​GAC​TTG​TGA​A
Human GAPDH	Forward	GCA​CCG​TCA​AGG​CTG​AGA​AC
	Reverse	TGG​TGA​AGA​CGC​CAG​TGG​A
Human TNF-α	Forward	CTG​CCT​GCT​GCA​CTT​TGG​AG
	Reverse	ACA​TGG​GCT​ACA​GGC​TTG​TCA​CT
Human IL-6	Forward	AAG​CCA​GAG​CTG​TGC​AGA​TGA​GTA
	Reverse	TGT​CCT​GCA​GCC​ACT​GGT​TC
Human IL-1β	Forward	CCA​GGG​ACA​GGA​TAT​GGA​GCA
	Reverse	TTC​AAC​ACG​CAG​GAC​AGG​TAC​AG

### 2.12 Western blot assay

Proteins were separated by SDS-PAGE and transferred onto PVDF membranes. The membranes were blocked and subsequently incubated with specific primary antibodies, followed by appropriate horseradish peroxidase–conjugated secondary antibodies. Protein bands were visualized using enhanced chemiluminescence (ECL). Representative bands are shown in the main figures. Western blot images were cropped for clarity and presentation purposes only, and the original, uncropped full-length blots are provided as Supplementary Material.

### 2.13 Statistical methods

GraphPad Prism v10.1.2 was used for statistical analyses and graph generation. All data are expressed as mean ± SEM. One-way ANOVA analyzed multi-group differences, followed by Levene’s test for variance homogeneity; Tukey’s post-hoc test was used for pairwise comparisons if ANOVA was significant. A p-value <0.05 was considered statistically significant.

## 3 Results

### 3.1 Rosacea target acquisition and Vicenin-2-rosacea target prediction

**FIGURE 1:**
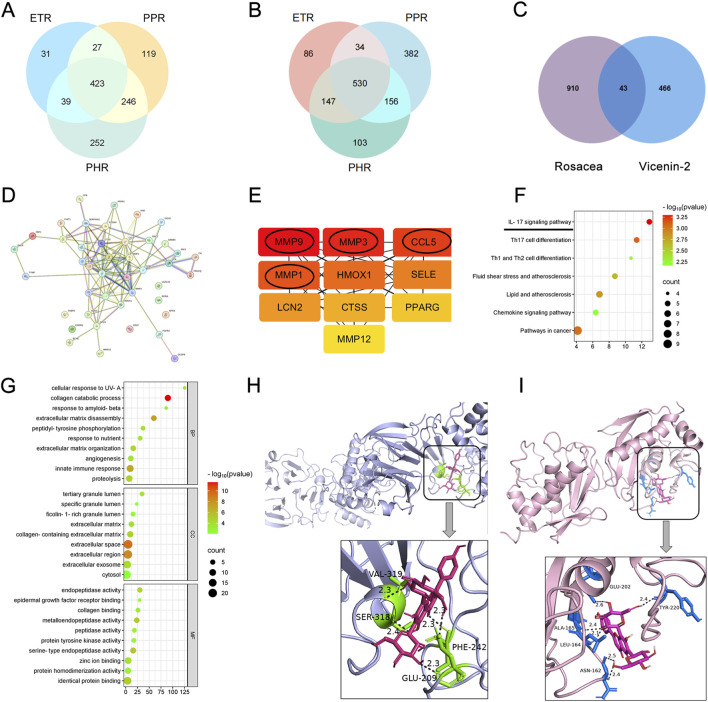
Target prediction of Vicenin-2 for the treatment of rosacea. **(A)** Common upregulated differentially expressed genes (DEGs) across different rosacea subtypes. **(B)** Common downregulated DEGs across different rosacea subtypes. **(C)** Identification of putative therapeutic targets of Vicenin-2 for rosacea using Venny 2.1. **(D)** Protein–protein interaction (PPI) network of the putative targets. **(E)** Top 10 hub genes screened based on the maximal clique centrality (MCC) algorithm. **(F)** Bubble plot of KEGG pathway enrichment analysis of the 43 putative targets. **(G)** Bubble plot of Gene Ontology (GO) enrichment analysis of the 43 putative targets. **(H)** Molecular docking model of Vicenin-2 with MMP1. **(I)** Molecular docking model of Vicenin-2 with MMP3.

The GEO database (GPL570 microarray platform) contains gene expression data from 19 rosacea lesion tissues and 10 healthy skin tissues (Buhl et al., 2015). Using the GEO2R tool, the number of DEGs in each rosacea subtype was analyzed: ETR had 1,317 DEGs; PPR had 1,917 DEGs; and PhR had 1,896 DEGs. The overlapping DEGs among the subtypes included 423 upregulated genes (Figure 1A) and 530 downregulated genes (Figure 1B). By querying public databases, 509 predicted targets of Vicenin-2 were obtained. Forty-three possible targets for Vicenin-2 in the treatment of rosacea were found by intersecting these databases (Figure 1C).

### 3.2 PPI network construction and core target prediction

A PPI network was constructed from the STRING database using *Homo sapiens* as the target species, with a confidence threshold of >0.4. Unconnected nodes were hidden to focus on the protein interaction network (Figure 1D). Among the 48 proteins encoded by potential genes, 225 interactions were identified, with an average node degree of 9.38 and a highly significant PPI enrichment p-value (<1.0e-16). The top 10 genes, selected using the CytoHubba tool with MCC scores, were identified as key targets for Vicenin-2 treatment of rosacea, including MMP9, MMP3, CCL5, and MMP1 (Figure 1E).

### 3.3 KEGG and GO enrichment analysis of potential targets

KEGG enrichment analysis showed that the potential targets were primarily enriched in immune-inflammatory signaling pathways, including IL-17, Th17 cell differentiation, and Th1/Th2 differentiation. Additionally, these genes were linked to lipid metabolism and atherosclerosis (Figure 1F). GO enrichment analysis indicated their involvement in key biological processes, including extracellular matrix breakdown and modulation of the innate immune response (Figure 1G). These findings suggest that the core targets may mediate their functions through these pathways.

### 3.4 Molecular docking

Molecular docking of the four key genes with Vicenin-2 showed binding energies of MMP1 (−9.2 kcal/mol), MMP3 (−8.2 kcal/mol), MMP9 (−7.6 kcal/mol), and CCL5 (−5.8 kcal/mol), indicating stronger binding affinity for MMP1 and MMP3. The docking results for these two proteins are shown in Figures 1H,I.

### 3.5 Topical application of Vicenin-2 solution dose-dependently alleviated LL-37-induced rosacea-like dermatitis in mice

**FIGURE 2:**
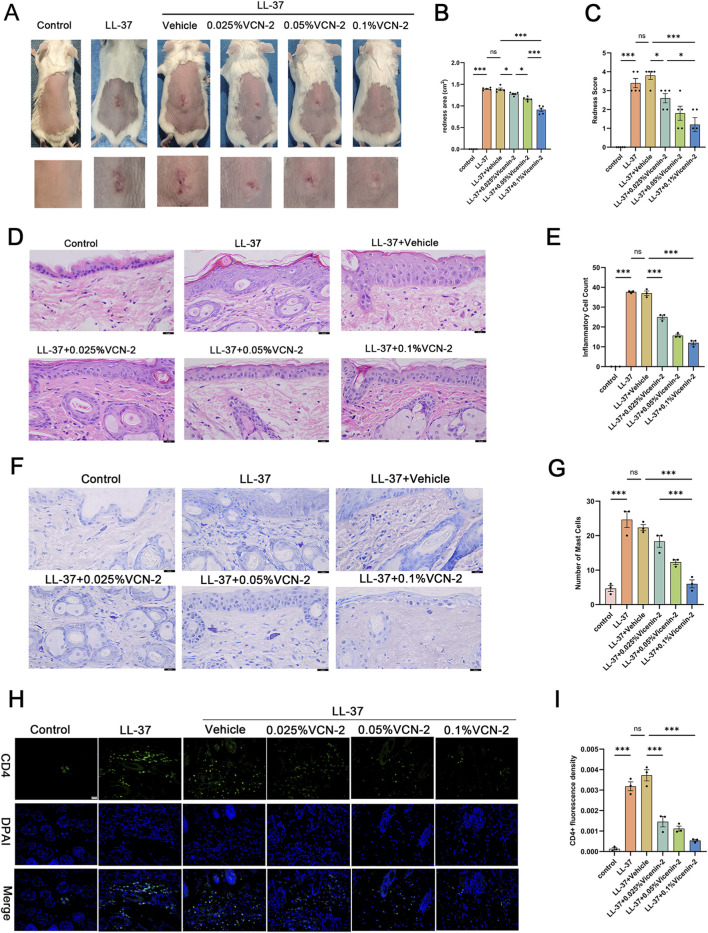
Topical Vicenin-2 solution ameliorates rosacea-like dermatitis phenotypes in mice. **(A)** Representative images of dorsal skin lesions in mice (n = 5). **(B)** Quantification of erythema area on the dorsal skin (cm^2^) (n = 5). **(C)** Erythema severity scores of dorsal lesions (n = 5). **(D)** Hematoxylin and eosin (H&E) staining of dorsal skin tissue: nuclei stained blue, and cytoplasm, muscle fibers, collagen fibers, and red blood cells stained in varying degrees of red (400×) (n = 3). **(E)** Quantitative analysis of inflammatory cell counts based on H&E staining at ×400 magnification. **(F)** Toluidine blue staining of dorsal skin tissue: mast cells stained purple-red (400×) (n = 3). **(G)** Quantitative analysis of mast cell counts based on toluidine blue staining at ×400 magnification. **(H)** Immunofluorescence staining of CD4^+^ T cells in dorsal skin tissue: nuclei stained blue with DAPI, and green fluorescence indicates positive signals (n = 3). **(I)** Quantification of fluorescence intensity of CD4^+^ T-cell-positive areas based on immunofluorescence staining. Statistical significance was analyzed using one-way ANOVA followed by Tukey’s post-hoc test. *p < 0.05, **p < 0.01, ***p < 0.001; ns, not significant. Abbreviation: VCN-2, Vicenin-2.

To explore Vicenin-2’s potential for treating rosacea, we created an LL-37-mediated rosacea-like dermatitis model. Results showed erythema and capillary dilation in the LL-37 and LL-37 + vehicle groups, while Vicenin-2 treatment reduced LL-37-induced erythema in a dose-dependent manner (Figure 2A). Quantitative analysis indicated a 34.17% decrease in erythema area in the 0.1% Vicenin-2 group (p < 0.001) relative to the LL-37 + vehicle group (Figure 2B). Blinded severity scoring also showed a 2.6-point decrease in erythema score for the 0.1% Vicenin-2 group (p < 0.001) (Figure 2C).

Histopathological analysis confirmed the anti-inflammatory effects of Vicenin-2. HE staining showed LL-37-induced epidermal hyperplasia and dermal inflammation, which were alleviated by Vicenin-2 in a dose-dependent manner (p < 0.001) (Figure 2D). Inflammatory cell counts are quantified in Figure 2E. Toluidine blue staining revealed dose-dependent inhibition of mast cell infiltration by Vicenin-2 (p < 0.001) (Figure 2F). Mast cell counts are summarized in Figure 2G. Immunofluorescence staining showed increased CD4^+^ T cell infiltration after LL-37 stimulation, which was inhibited by Vicenin-2 (p < 0.001) (Figure 2H). Quantitative analysis of immunofluorescence staining is shown in Figure 2I.

### 3.6 Vicenin-2 suppresses inflammatory mediator production in lesional skin

**FIGURE 3:**
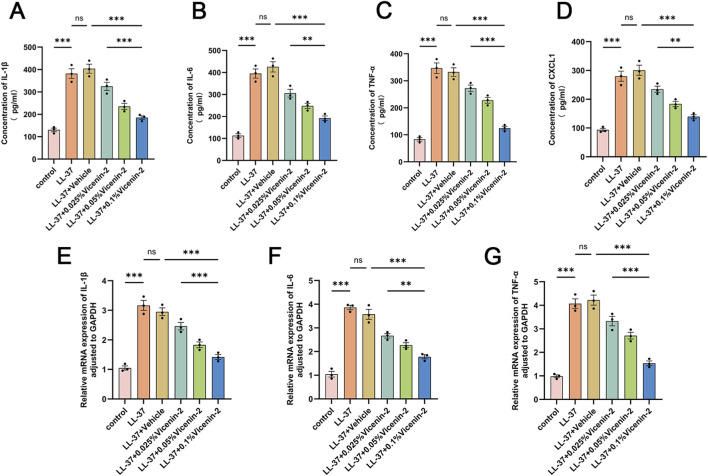
Topical Vicenin-2 solution reduces levels of inflammatory cytokines in mouse skin lesions. **(A–D)** Levels of IL-1β, IL-6, TNF-α, and CXCL1 in mouse lesion tissues measured by ELISA. **(E–G)** mRNA expression levels of IL-1β, IL-6, and TNF-α in lesion tissues were measured by quantitative real-time PCR (RT-qPCR). Statistical significance was analyzed using one-way ANOVA followed by Tukey’s post-hoc test. *p < 0.05, **p < 0.01, ***p < 0.001; ns, not significant.

Based on histological findings, we analyzed key inflammatory mediators in skin lesions. ELISA showed that LL-37 injection significantly increased IL-1β, IL-6, TNF-α, and CXCL1 protein levels in mouse skin tissue (Figures 3A–D, p < 0.001 vs. control). Vicenin-2 concentration-dependently reduced these levels (p < 0.001). RT-qPCR confirmed that Vicenin-2 dose-dependently decreased LL-37-induced mRNA levels of IL-1β, IL-6, and TNF-α (P < 0.001 vs. LL-37 + vehicle, Figures 3E–G). Thus, Vicenin-2 downregulated inflammatory mediators at both protein and mRNA levels.

### 3.7 Vicenin-2 inhibits IL-17RA signaling cascade and suppresses NF-κB pathway activation *in vivo*

**FIGURE 4:**
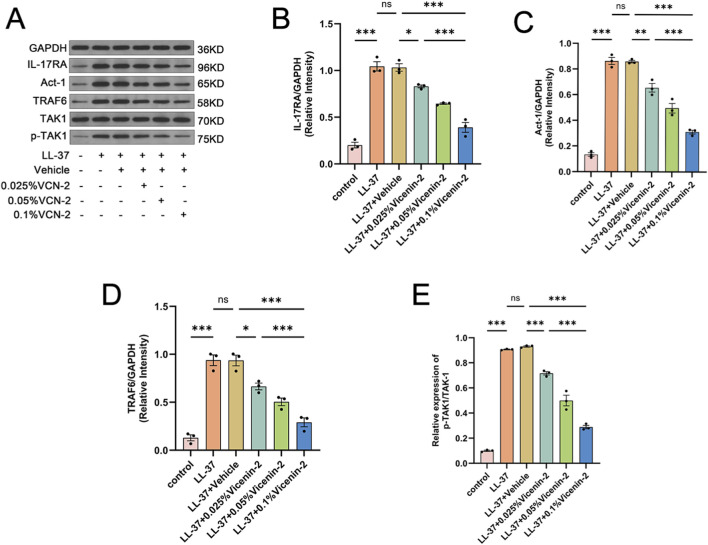
Topical Vicenin-2 solution suppresses IL-17RA signaling transduction in mouse skin tissue. **(A)** Immunoblot analysis of key proteins involved in IL-17RA signaling in mouse dorsal skin tissue. **(B)** Relative protein expression level of IL-17RA normalized to GAPDH. **(C)** Relative protein expression level of ACT1 normalized to GAPDH. **(D)** Relative protein expression level of TRAF6 normalized to GAPDH. **(E)** Relative phosphorylation level of TAK1 (p-TAK1/TAK1). Statistical significance was analyzed using one-way ANOVA followed by Tukey’s post-hoc test. *p < 0.05, **p < 0.01, ***p < 0.001; ns, not significant. The Western blot images shown were cropped and assembled for clarity and presentation purposes. The original, uncropped full-length blots are provided in the Supplementary Material.

**FIGURE 5:**
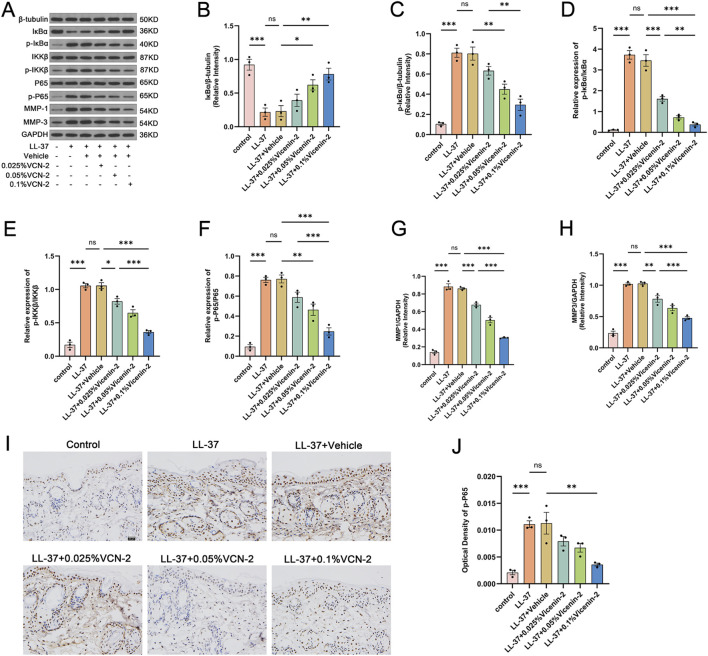
Vicenin-2 inhibits activation of the NF-κB signaling pathway in mouse lesion tissues. **(A)** Immunoblot analysis of key proteins in the NF-κB signaling pathway and downstream effectors in mouse dorsal skin tissue (n = 3). **(B)** Relative protein expression level of IκBα normalized to β-Tubulin. **(C)** Relative protein expression level of phosphorylated IκBα (p-IκBα) normalized to β-Tubulin. **(D)** Relative phosphorylation level of IκBα (p-IκBα/IκBα). **(E)** Relative phosphorylation level of IKKβ (p-IKKβ/IKKβ). **(F)** Relative phosphorylation level of p65 (p-p65/p65). **(G)** Relative protein expression level of MMP1 normalized to GAPDH. **(H)** Relative protein expression level of MMP3 normalized to GAPDH. **(I)** Immunohistochemical staining showing nuclear translocation of p-p65 in mouse skin tissue: nuclei stained blue, and brown/yellow-brown signals indicate p-p65 (400×). **(J)** Quantification of integrated optical density (IOD) based on immunohistochemical staining. Statistical significance was analyzed using one-way ANOVA followed by Tukey’s post-hoc test. *p < 0.05, **p < 0.01, ***p < 0.001; ns, not significant. The Western blot images shown were cropped and assembled for clarity and presentation purposes. The original, uncropped full-length blots are provided in the Supplementary Material.

Building on our network pharmacology and KEGG enrichment findings, we investigated IL-17 signaling via Western blotting. Vicenin-2 diminished the expression of IL-17RA, ACT1, and TRAF6 in murine cutaneous lesions and inhibited TAK1 phosphorylation in a dose-dependent manner (Figures 4A–E, p < 0.05). Molecular docking results led us to examine NF-κB activation and MMP1/3 expression. Western blotting demonstrated that Vicenin-2 suppressed LL-37-induced levels of p-IKKβ and p-p65 while not influencing total IKKβ and p65 levels (p < 0.05 vs. LL-37+Vehicle). LL-37 diminished overall IκBα expression, whereas Vicenin-2 reinstated IκBα levels and decreased p-IκBα levels, thereby inhibiting NF-κB activation (Figures 5A–F). Additionally, Vicenin-2 reduced MMP1/3 expression (Figures 5G,H, p < 0.01). Immunohistochemistry showed that Vicenin-2 dose-dependently inhibited LL-37-induced nuclear translocation of p-p65 (Figures 5I,J). These findings indicate that Vicenin-2 may inhibit IL-17RA signaling and attenuate NF-κB activation, thereby exerting anti-inflammatory and anti-matrix-remodeling effects *in vivo*.

### 3.8 Vicenin-2 attenuates LL-37-induced inflammatory responses in HaCaT cells with minimal cytotoxicity

**FIGURE 6:**
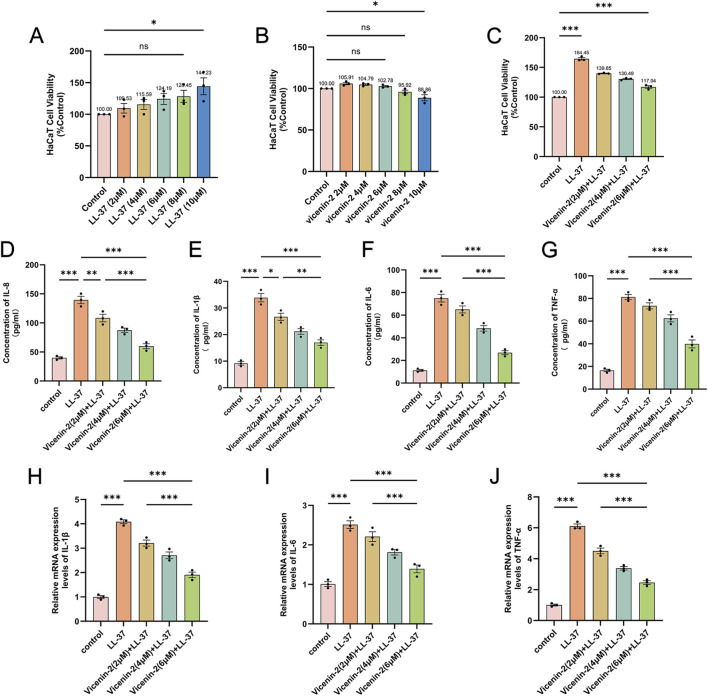
Vicenin-2 attenuates LL-37–induced inflammatory responses in HaCaT cells. **(A)** Determination of the non-cytotoxic concentration range of LL-37 using the CCK-8 assay. **(B)** Determination of the non-cytotoxic concentration range of Vicenin-2 using the CCK-8 assay. **(C)** Cell viability of the LL-37–induced HaCaT inflammatory model after Vicenin-2 treatment, assessed by the CCK-8 assay. **(D–G)** Protein levels of IL-8, IL-1β, IL-6, and TNF-α in HaCaT cell culture supernatants were measured by ELISA. **(H–J)** mRNA expression levels of IL-1β, IL-6, and TNF-α in HaCaT cells measured by RT-qPCR. Statistical significance was analyzed using one-way ANOVA followed by Tukey’s post-hoc test. *p < 0.05, **p < 0.01, ***p < 0.001; ns, not significant.

To explore Vicenin-2’s anti-inflammatory mechanisms *in vitro*, we first determined its safe concentration range using CCK-8 assays. Results showed that 10 μM LL-37 did not affect cell viability (Figure 6A), and Vicenin-2 ≤6 μM had no significant impact on HaCaT cell viability after LL-37 treatment (Figures 6B,C). Based on this, we selected 2, 4, and 6 μM for further experiments. ELISA analysis showed that LL-37 significantly increased TNF-α, IL-8, IL-1β, and IL-6 levels (p < 0.001), while Vicenin-2 pretreatment dose-dependently inhibited these cytokines, with 6 μM achieving a 49%–64% inhibition (p < 0.001 vs. LL-37) (Figures 6D–G). RT-qPCR confirmed that Vicenin-2 reduced TNF-α, IL-1β, and IL-6 mRNA expression by 44%–59% at 6 μM (p < 0.001) (Figures 6H–J). These results suggest a transcriptional regulatory mechanism for Vicenin-2’s broad anti-inflammatory effects, with excellent safety.

### 3.9 Vicenin-2 inhibits IL-17RA signaling cascade and NF-κB pathway activation *in vitro*

**FIGURE 7:**
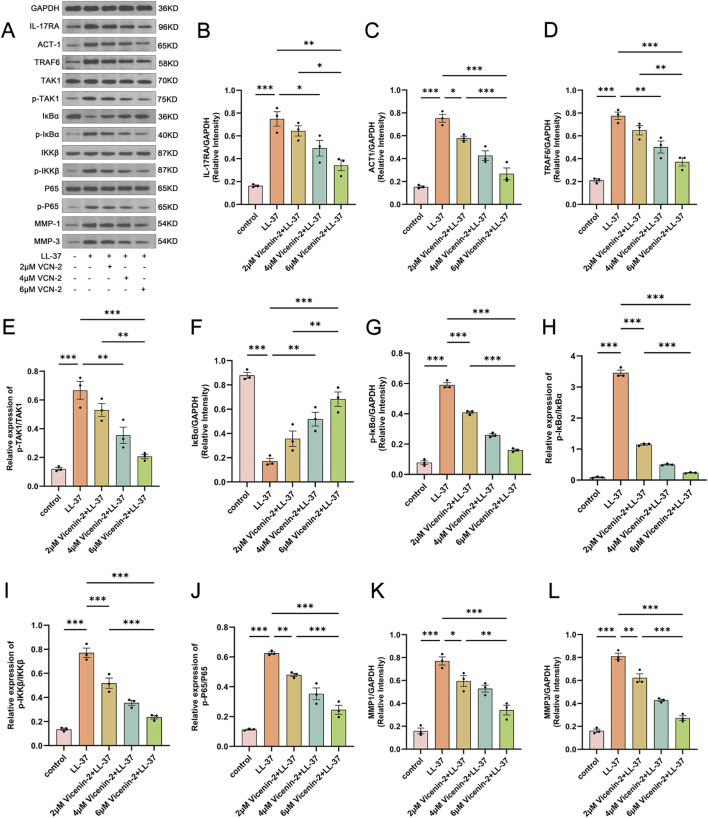
Vicenin-2 suppresses IL-17RA signaling transduction and NF-κB activation in the HaCaT inflammatory model. **(A)** Immunoblot analysis of key proteins involved in IL-17RA signaling and NF-κB activation, as well as downstream effectors, in HaCaT cells. **(B)** Relative protein expression level of IL-17RA normalized to GAPDH. **(C)** Relative protein expression level of ACT1 normalized to GAPDH. **(D)** Relative protein expression level of TRAF6 normalized to GAPDH. **(E)** Relative phosphorylation level of TAK1 (p-TAK1/TAK1). **(F)** Relative protein expression level of IκBα normalized to GAPDH. **(G)** Relative protein expression level of p-IκBα normalized to GAPDH. **(H)** Relative phosphorylation level of IκBα (p-IκBα/IκBα). **(I)** Relative phosphorylation level of IKKβ (p-IKKβ/IKKβ). **(J)** Relative phosphorylation level of p65 (p-p65/p65). **(K)** Relative protein expression level of MMP1 normalized to GAPDH. **(L)** Relative protein expression level of MMP3 normalized to GAPDH. Statistical significance was analyzed using one-way ANOVA followed by Tukey’s post-hoc test. *p < 0.05, **p < 0.01, ***p < 0.001; ns, not significant. The Western blot images shown were cropped and assembled for clarity and presentation purposes. The original, uncropped full-length blots are provided in the Supplementary Material.

To eliminate interference from the immune microenvironment, we validated relevant pathway expression in an HaCaT cell inflammation model (Figure 7A). Western blot results showed Vicenin-2 dose-dependently suppressed IL-17RA, downregulated ACT1 and TRAF6, and reduced TAK1 phosphorylation compared to LL-37 (Figures 7B–E, p < 0.05). Additionally, LL-37 decreased IκBα and increased p-IκBα, while Vicenin-2 restored IκBα and inhibited p-IκBα expression (Figures 7F–J). Vicenin-2 also reduced p-IKKβ and p-p65 expression levels and downregulated MMP1/3 secretion compared to LL-37 (Figures 7K,L, p < 0.01). These results, showing a dose-response effect, align with our *in vivo* findings. In conclusion, Vicenin-2 alleviates LL-37-induced inflammation in both the mouse and HaCaT cell models, likely by inhibiting IL-17RA signaling and reducing NF-κB pathway activation.

## 4 Discussion

Rosacea, a chronic, recurrent disease, involves various pathogenic factors and significantly affects patients. Despite extensive research efforts, existing treatments remain suboptimal. This study used computational biology tools to elucidate the therapeutic efficacy and mechanisms of action of the flavonoid Vicenin-2 for the treatment of rosacea, combining *in vitro* and animal models.

Network pharmacology predicted that Vicenin-2 targets MMP9, MMP3, CCL5, and MMP1 in rosacea, with molecular docking showing strong binding to MMP3 and MMP1. GO and KEGG analyses revealed that Vicenin-2 regulates processes like collagen degradation, extracellular matrix disassembly, the innate immune response, IL-17 signaling, and Th17 cell differentiation, all linked to rosacea onset and progression (Buhl et al., 2015; Amir et al., 2019; Hayran et al., 2022). IL-17-positive T helper cells and other associated immune cells are more frequently infiltrated into the skin lesions of rosacea patients, indicating strong activation of the IL-17 signaling pathway (Buhl et al., 2015). Collagen degradation is a core step in extracellular matrix disassembly (D'Armiento, 2002). In rosacea lesions, upregulated MMPs drive collagen degradation and extracellular matrix remodeling, loosening the dermal matrix and altering the perivascular microenvironment, thereby promoting vasodilation and vascular phenotypes such as telangiectasia (Schwab et al., 2011; Helfrich et al., 2015). Among these enzymes, MMP-1-mediated degradation of type I collagen has been considered a key pro-angiogenic signal (Varani et al., 2008). In rosacea, hyperactivated innate immunity and upregulation of pattern recognition receptors, such as TLR2, make the skin overly responsive to microbial and damage signals, triggering the KLK5-cathelicidin pathway and increased LL-37 production (Andrusiewicz et al., 2025; Yamasaki et al., 2011).

IL-17, a crucial pro-inflammatory cytokine produced by Th17 cells, plays a key role in the onset and progression of many chronic inflammatory diseases (Beringer et al., 2016). IL-17RA, a type I transmembrane protein, acts as a common receptor subunit for IL-17 family members like IL-17A, IL-17E, and IL-17A/F heterodimers (Krstic et al., 2015). IL-17A binds to IL-17RA on target cells such as keratinocytes and epithelial cells, initiating downstream signaling. IL-17RA must form heterodimers with other receptor subunits, such as IL-17RC, to complete signal transduction (Goepfert et al., 2022). The IL-17A/IL-17RA/IL-17RC receptor activates downstream adaptor proteins, such as ACT1, which recruits effector proteins, including TAK1 and IKKα/IKKβ, through K63-linked polyubiquitination of TRAF6, ultimately activating the NF-κB pathway (Amatya et al., 2017; Li X. et al., 2019; Draberova et al., 2020). A recent review of small-scale IL-17 inhibitor studies in rosacea showed a 100% response rate but noted adverse events, including infections. Further clinical research is needed for a more comprehensive evaluation (Dai et al., 2024; Kumar et al., 2020). IL-17A-neutralizing antibodies have been shown in an *in vivo* investigation to improve angiogenesis, lower inflammation, and lessen fibrosis in rosacea lesions, while also suppressing the CXCL5/CXCR2 axis, thereby slowing disease progression (Zhang et al., 2024). Targeting IL-17A or inhibiting downstream signaling may be a promising rosacea treatment. Our experiments showed that topical Vicenin-2 reduced LL-37-induced inflammation in mice and HaCaT cells by dampening the IL-17RA/ACT1/TRAF6 axis and downregulating TAK1 phosphorylation.

Pro-inflammatory cytokines activate the IKK complex, leading to IκB degradation and NF-κB (p65/p50) translocation to the nucleus, where it initiates transcription of target genes (Tornatore et al., 2012). Studies suggest that NF-κB activation increases IL-17RA expression, creating a positive feedback loop that amplifies and sustains skin inflammation, contributing to the chronicity of rosacea (Wei et al., 2021). Our preclinical findings suggest that Vicenin-2 may reverse LL-37-induced IκBα degradation and suppress p-IκBα, p-IKKβ, and p-p65 expression, inhibiting pathway activation. MMP1 and MMP3, key downstream effectors, degrade the extracellular matrix, regulate inflammation, and exacerbate skin barrier disruption in chronic inflammatory skin diseases (Port et al., 2025). Our study showed that Vicenin-2 dose-dependently inhibited LL-37-induced MMP1 and MMP3 expression, suggesting its role in tissue repair and collagen remodeling during rosacea inflammation.

Levels of pro-inflammatory cytokines, including TNF-α, IL-1β, IL-8, and IL-6, are elevated in rosacea skin samples. These cytokines not only directly cause inflammation and vascular responses but also recruit and activate CD4^+^ T cells (Yang et al., 2024; Li Y. et al., 2019). Research has confirmed significant CD4^+^ T cell infiltration in rosacea lesions, which is also observed in LL-37-induced mouse skin lesions (Qing et al., 2025; Jia et al., 2025). Immunohistochemistry showed that Vicenin-2 reduced LL-37-induced CD4^+^ T cell infiltration in mouse skin in a dose-dependent manner, indicating its potential for immune regulation in rosacea. Additionally, Vicenin-2 decreased both inflammatory cell infiltration and dermal mast cell density, suggesting its anti-inflammatory effects may involve inhibiting mast cell-mediated immune responses.

Vicenin-2’s favorable safety profile and strategic advantages over existing therapies further enhance its clinical translation potential. Current first-line topical treatments include metronidazole, which alleviates inflammation through a single mechanism: inhibiting neutrophil chemotaxis and activity (Volk et al., 2025). Azelaic acid reduces inflammation and oxidative stress by inhibiting ROS, downregulating cytokines, and reducing serine protease activity to improve keratinocyte differentiation, though it may cause skin irritation in some patients (Petrovici et al., 2025). Ivermectin directly targets Demodex mites and has potent anti-inflammatory effects but shows limited efficacy in ETR (Ebbelaar et al., 2018; Schaller et al., 2017). In contrast, Vicenin-2’s multi-target inhibition of IL-17RA/NF-κB and MMP1/3 offers a disease-modifying approach rather than merely symptomatic relief. *In vitro*, Vicenin-2 showed no cytotoxicity up to 6 μM and was effective topically at 0.1% in mice without irritation, suggesting a broad therapeutic window. This makes it a promising alternative for managing recurrent rosacea. Future development should focus on optimizing formulations, such as nanoemulsion delivery, to improve dermal bioavailability.

Despite promising results, our study has limitations. First, while we validated Vicenin-2’s effects in cellular and animal models, the absence of validation in IL-17RA overexpression or knockout models limits our understanding of its mechanism. Second, our study focused on the short-term topical application of Vicenin-2. Although no significant irritation or cytotoxicity was observed, long-term efficacy and safety remain unclear. Then, although our conclusions are grounded in robust molecular analysis and *in vivo* phenotypical data, visual evidence of cellular phenotypic shifts would further strengthen the narrative. This aspect will be addressed in subsequent research to complement our current molecular findings and enhance the visual clarity of the cellular transformations involved. Additionally, despite the absence of detectable adverse reactions, including cytotoxicity or skin irritation, and the negligible concentration of DMSO employed, incorporating a vehicle control group in future investigations will be essential to rigorously validate the specific therapeutic impact of Vicenin-2. Finally, the short-term inflammatory model used cannot fully assess Vicenin-2’s long-term effects. Future research could involve the establishment of chronic rosacea inflammation models and the implementation of multi-model validations including fibroblasts, along with the inclusion of positive control groups, to further consolidate our experimental results. These limitations will be key areas for our future research.

In summary, our preclinical results show that Vicenin-2 may alleviate LL-37-induced skin inflammation resembling rosacea, likely by modulating IL-17RA/NF-κB signaling to suppress inflammation and regulate immunity. These findings suggest that Vicenin-2 has potential as an alternative rosacea treatment.

## Data Availability

The original contributions presented in the study are included in the article/Supplementary Material, further inquiries can be directed to the corresponding author.

## References

[B1] Amatya N. Garg A. V. Gaffen S. L. (2017). IL-17 signaling: the yin and the yang. Trends Immunol. 38 (5), 310–322. 10.1016/j.it.2017.01.006 28254169 PMC5411326

[B2] Amir A. A. Vender R. Vender R. (2019). The role of IL-17 in papulopustular rosacea and future directions. J. Cutan. Med. Surg. 23 (6), 635–641. 10.1177/1203475419867611 31402691

[B3] Andrusiewicz A. Khimuk S. Mijas D. Shmorhun B. Nowicka D. (2025). Molecular mechanisms in the etiopathology of rosacea-systematic review. Int. J. Mol. Sci. 26 (23), 11292. 10.3390/ijms262311292 41373451 PMC12692705

[B4] Beringer A. Noack M. Miossec P. (2016). IL-17 in chronic inflammation: from discovery to targeting. Trends Mol. Med. 22 (3), 230–241. 10.1016/j.molmed.2016.01.001 26837266

[B5] Buhl T. Sulk M. Nowak P. Buddenkotte J. Mcdonald I. Aubert J. (2015). Molecular and morphological characterization of inflammatory infiltrate in rosacea reveals activation of Th1/Th17 pathways. J. Invest. Dermatol. 135 (9), 2198–2208. 10.1038/jid.2015.141 25848978

[B6] Clanner-Engelshofen B. M. Bernhard D. Dargatz S. Flaig M. J. Gieler U. Kinberger M. (2022). S2k guideline: rosacea. J. Dtsch. Dermatol. Ges. 20 (8), 1147–1165. 10.1111/ddg.14849 35929658

[B7] Crawford G. H. Pelle M. T. James W. D. (2004). Rosacea: I. Etiology, pathogenesis, and subtype classification. J. Am. Acad. Dermatol. 51 (3), 327–341. 10.1016/j.jaad.2004.03.030 15337973

[B8] D'Armiento J. (2002). Matrix metalloproteinase disruption of the extracellular matrix and cardiac dysfunction. Trends Cardiovasc. Med. 12 (3), 97–101. 10.1016/s1050-1738(01)00160-8 12007733

[B9] Dai X. Zhang C. Yin Z. (2024). Therapy outcomes of IL-17 and JAK inhibitors in rosacea: a systematic review. J. Biomed. Res. 39 (3), 317–318. 10.7555/JBR.38.20240107 39164193 PMC12239976

[B10] Del R. J. Gallo R. L. Kircik L. Thiboutot D. Baldwin H. E. Cohen D. (2012). Why is rosacea considered to be an inflammatory disorder? The primary role, clinical relevance, and therapeutic correlations of abnormal innate immune response in rosacea-prone skin. J. Drugs Dermatol. 11 (6), 694–700. 22648215

[B11] Draberova H. Janusova S. Knizkova D. Semberova T. Pribikova M. Ujevic A. (2020). Systematic analysis of the IL-17 receptor signalosome reveals a robust regulatory feedback loop. EMBO. J. 39 (17), e104202. 10.15252/embj.2019104202 32696476 PMC7459424

[B12] Duan X. Wu T. Liu T. Yang H. Ding X. Chen Y. (2019). Vicenin-2 ameliorates oxidative damage and photoaging via modulation of MAPKs and MMPs signaling in UVB radiation exposed human skin cells. J. Photochem. Photobiol. B 190, 76–85. 10.1016/j.jphotobiol.2018.11.018 30502588

[B13] Ebbelaar C. Venema A. W. Van Dijk M. R. (2018). Topical ivermectin in the treatment of papulopustular rosacea: a systematic review of evidence and clinical Guideline recommendations. Dermatol. Ther. 8 (3), 379–387. 10.1007/s13555-018-0249-y 29943217 PMC6109029

[B14] Feng C. Zhang H. Wang P. Zhang L. Liu X. Yan G. (2024). Oroxylin A suppress LL-37 generated rosacea-like skin inflammation through the modulation of SIRT3-SOD2-NF-κB signaling pathway. Int. Immunopharmacol. 129, 111636. 10.1016/j.intimp.2024.111636 38364746

[B15] Galvao J. Davis B. Tilley M. Normando E. Duchen M. R. Cordeiro M. F. (2014). Unexpected low-dose toxicity of the universal solvent DMSO. FASEB. J. 28 (3), 1317–1330. 10.1096/fj.13-235440 24327606

[B16] Gether L. Overgaard L. K. Egeberg A. Thyssen J. P. (2018). Incidence and prevalence of rosacea: a systematic review and meta-analysis. Br. J. Dermatol. 179 (2), 282–289. 10.1111/bjd.16481 29478264

[B17] Goepfert A. Barske C. Lehmann S. Wirth E. Willemsen J. Gudjonsson J. E. (2022). IL-17-induced dimerization of IL-17RA drives the formation of the IL-17 signalosome to potentiate signaling. Cell Rep. 41 (3), 111489. 10.1016/j.celrep.2022.111489 36260993 PMC9637376

[B18] Grafanaki K. Bakoli S. D. Maniatis A. Pasmatzi E. (2025). The exposomal imprint on rosacea: more than skin deep. J. Eur. Acad. Dermatol. Venereol. 40 (3), 387–403. 10.1111/jdv.70112 41081484 PMC12933707

[B19] Guo B. Wu M. Tong L. Yu J. Liu R. Deng M. (2025). Hydroxysafflower yellow a protects against UVA- and UVB-induced skin aging by suppressing cell apoptosis and SASP via targeting JNK and p38 MAPK pathway. J. Photochem. Photobiol. B 274, 113340. 10.1016/j.jphotobiol.2025.113340 41421060

[B20] Hayran Y. _en O. Fırat O. E. Yücel Ç. Eren F. Külcü Ç. S. (2022). Serum IL-17 levels in patients with rosacea. J. Cosmet. Dermatol. 21 (3), 1147–1153. 10.1111/jocd.14169 33877738

[B21] Helfrich Y. R. Maier L. E. Cui Y. Fisher G. J. Chubb H. Fligiel S. (2015). Clinical, histologic, and molecular analysis of differences between erythematotelangiectatic rosacea and telangiectatic photoaging. JAMA Dermatol 151 (8), 825–836. 10.1001/jamadermatol.2014.4728 25798811

[B22] Hu X. Chen M. Tan B. Yang H. Li S. Li R. (2025). Vicenin-2 reduces inflammation and apoptosis to relieve skin photoaging via suppressing GSK3β. J. Photochem. Photobiol. B Biol. 264, 113117. 10.1016/j.jphotobiol.2025.113117 39923642

[B23] Jia T. Xia Y. Yi M. Zhang X. Zheng Y. Che D. (2025). Casticin reduces rosacea-related inflammation by inhibiting mast cell activation via Mas-related G protein-coupled receptor X2. Inflammopharmacology 33 (4), 1935–1947. 10.1007/s10787-025-01639-8 39821787

[B24] Jiang Y. Tsoi L. C. Billi A. C. Ward N. L. Harms P. W. Zeng C. (2020). Cytokinocytes: the diverse contribution of keratinocytes to immune responses in skin. JCI Insight 5 (20), e142067. 10.1172/jci.insight.142067 33055429 PMC7605526

[B25] Kang H. Ku S. K. Jung B. Bae J. S. (2015). Anti-inflammatory effects of vicenin-2 and scolymoside *in vitro* and *in vivo* . Inflamm. Res. 64 (12), 1005–1021. 10.1007/s00011-015-0886-x 26482935

[B26] Krstic J. Obradovic H. Kukolj T. Mojsilovic S. Okic-Dordevic I. Bugarski D. (2015). An overview of Interleukin-17A and Interleukin-17 receptor A structure, interaction and signaling. Protein pept. Lett 22 (7), 570–578. 10.2174/0929866522666150520145554 25990083

[B27] Kumar A. M. Chiou A. S. Shih Y. H. Li S. Chang A. (2020). An exploratory, open-label, investigator-initiated study of interleukin-17 blockade in patients with moderate-to-severe papulopustular rosacea. Br. J. Dermatol. 183 (5), 942–943. 10.1111/bjd.19172 32364247

[B28] Levin M. Romano T. Matassa K. De Guise S. (2014). Validation of a commercial canine assay kit to measure pinniped cytokines. Vet. Immunol. Immunopathol. 160 (1-2), 90–96. 10.1016/j.vetimm.2014.04.001 24845148

[B29] Li X. Bechara R. Zhao J. Mcgeachy M. J. Gaffen S. L. (2019). IL-17 receptor-based signaling and implications for disease. Nat. Immunol. 20 (12), 1594–1602. 10.1038/s41590-019-0514-y 31745337 PMC6943935

[B30] Li Y. Xie H. Deng Z. Wang B. Tang Y. Zhao Z. (2019). Tranexamic acid ameliorates rosacea symptoms through regulating immune response and angiogenesis. Int. Immunopharmacol. 67, 326–334. 10.1016/j.intimp.2018.12.031 30578968

[B31] Marrassini C. Davicino R. Acevedo C. Anesini C. Gorzalczany S. Ferraro G. (2011). Vicenin-2, a potential anti-inflammatory constituent of Urtica circularis. J. Nat. Prod. 74 (6), 1503–1507. 10.1021/np100937e 21608987

[B32] Meng X. Li Y. Wang F. Li T. Wang B. Wang Q. (2024). Quercetin attenuates inflammation in rosacea by directly targeting p65 and ICAM-1. Life Sci. 347, 122675. 10.1016/j.lfs.2024.122675 38688383

[B33] Petrovici A. G. Spennato M. Bîtcan I. Péter F. Cotarcă L. Todea A. (2025). A comprehensive review of Azelaic acid pharmacological properties, clinical applications, and innovative topical formulations. Pharmaceuticals 18 (9), 1273. 10.3390/ph18091273 41011144 PMC12472904

[B34] Port H. Andelic M. Nyström A. Haack A. M. Kalogeropoulos K. Nielsen V. W. (2025). Extracellular matrix alterations in inflammatory skin diseases: emerging biomarkers and clinical implications. Clin. Exp. Dermatol. 50 (11), 2118–2132. 10.1093/ced/llaf308 40632820

[B35] Qing Y. Wu J. Xu B. Xu Z. Ye S. Wang Y. (2025). DNAJB2 attenuates rosacea skin inflammation and angiogenesis by inhibiting the endoplasmic reticulum stress-mediated TLR2/Myd88/NF-κB pathway. Inflammation 48 (5), 3472–3486. 10.1007/s10753-025-02278-5 40035989 PMC12596359

[B36] Rainer B. M. Kang S. Chien A. L. (2017). Rosacea: epidemiology, pathogenesis, and treatment. Dermatoendocrinol 9 (1), e1361574. 10.1080/19381980.2017.1361574 29484096 PMC5821167

[B37] Sarkar R. Podder I. Jagadeesan S. (2020). Rosacea in skin of color: a comprehensive review. Indian J. Dermatol. Venereol. Leprol. 86 (6), 611–621. 10.4103/ijdvl.IJDVL_769_19 33109832

[B38] Schaller M. Gonser L. Belge K. Braunsdorf C. Nordin R. Scheu A. (2017). Dual anti-inflammatory and anti-parasitic action of topical ivermectin 1% in papulopustular rosacea. J. Eur. Acad. Dermatol. Venereol. 31 (11), 1907–1911. 10.1111/jdv.14437 28653460

[B39] Schwab V. D. Sulk M. Seeliger S. Nowak P. Aubert J. Mess C. (2011). Neurovascular and neuroimmune aspects in the pathophysiology of rosacea. J. Investig. Dermatol Symp. Proc. 15 (1), 53–62. 10.1038/jidsymp.2011.6 22076328 PMC3704331

[B40] Shin M. H. Kwon Y. Lee S. Kim H. B. Kim H. Jeong S. (2025). Protective effect of a novel metal-phenolic network composite against ultraviolet-induced skin damage by modulating MAPK/AP-1/NF-κB signaling pathways and attenuating oxidative stress in human keratinocytes. Biomed. Pharmacother. 193, 118874. 10.1016/j.biopha.2025.118874 41352006

[B41] Thiboutot D. Anderson R. Cook-Bolden F. Draelos Z. Gallo R. L. Granstein R. D. (2020). Standard management options for rosacea: the 2019 update by the national rosacea society expert committee. J. Am. Acad. Dermatol. 82 (6), 1501–1510. 10.1016/j.jaad.2020.01.077 32035944

[B42] Tornatore L. Thotakura A. K. Bennett J. Moretti M. Franzoso G. (2012). The nuclear factor kappa B signaling pathway: integrating metabolism with inflammation. Trends Cell Biol. 22 (11), 557–566. 10.1016/j.tcb.2012.08.001 22995730

[B43] Varani J. Perone P. Warner R. L. Dame M. K. Kang S. Fisher G. J. (2008). Vascular tube formation on matrix metalloproteinase-1-damaged collagen. Br. J. Cancer. 98 (10), 1646–1652. 10.1038/sj.bjc.6604357 18443597 PMC2391116

[B44] Volk K. Ulfers A. Yi R. C. Feldman S. Taylor S. L. (2025). Treatment management for rosacea: current pharmacological and non-pharmacological options. Expert Rev. Clin. Pharmacol. 18 (8), 589–605. 10.1080/17512433.2025.2550727 40836652

[B45] Wang Y. Zhang Z. Cao L. Huang S. Huang X. Zhang Z. (2026). Therapeutic effects of the n-butanol extract of Potentilla freyniana bornm. in hepatocellular carcinoma cells. J. Ethnopharmacol. 354, 120492. 10.1016/j.jep.2025.120492 40902811

[B46] Wei X. Li C. Zhang Y. Li K. Li J. Ai K. (2021). Fish NF-κB couples TCR and IL-17 signals to regulate ancestral T-cell immune response against bacterial infection. FASEB. J. 35 (4), e21457. 10.1096/fj.202002393RR 33689192

[B47] Yamasaki K. Di Nardo A. Bardan A. Murakami M. Ohtake T. Coda A. (2007). Increased serine protease activity and cathelicidin promotes skin inflammation in rosacea. Nat. Med. 13 (8), 975–980. 10.1038/nm1616 17676051

[B48] Yamasaki K. Kanada K. Macleod D. T. Borkowski A. W. Morizane S. Nakatsuji T. (2011). TLR2 expression is increased in rosacea and stimulates enhanced serine protease production by keratinocytes. J. Invest. Dermatol. 131 (3), 688–697. 10.1038/jid.2010.351 21107351 PMC3085277

[B49] Yang F. Wang L. Song D. Zhang L. Wang X. Du D. (2024). Signaling pathways and targeted therapy for rosacea. Front. Immunol. 15, 1367994. 10.3389/fimmu.2024.1367994 39351216 PMC11439730

[B50] Zhang J. H. Zhang D. X. Zhao L. P. Yan T. T. Zhang Q. Jia J. Z. (2016). Effect of rapamycin on the migration of human epidermal cell line HaCaT and its possible molecular mechanism. Zhonghua Shao Shang Za Zhi 32 (1), 40–45. 10.3760/cma.j.issn.1009-2587.2016.01.011 27426069

[B51] Zhang C. Jin H. Kang Y. Wu Y. Zheng R. Zhang Z. (2024). IL-17A-neutralizing antibody ameliorates inflammation and fibrosis in rosacea by antagonizing the CXCL5/CXCR2 axis. FASEB. J. 38 (19), e70096. 10.1096/fj.202400006R 39370827

[B52] Zhao L. Zhang H. Li N. Chen J. Xu H. Wang Y. (2023). Network pharmacology, a promising approach to reveal the pharmacology mechanism of Chinese medicine formula. J. Ethnopharmacol. 309, 116306. 10.1016/j.jep.2023.116306 36858276

[B53] Zhou L. Zhong Y. Wang Y. Deng Z. Huang Y. Wang Q. (2023). EGCG identified as an autophagy inducer for rosacea therapy. Front. Pharmacol. 14, 1092473. 10.3389/fphar.2023.1092473 36937834 PMC10014537

[B54] Zhu F. Qu L. Xu R. Yuan Y. Zhang S. Chen Y. (2025). Synergistic anti-photoaging and anti-inflammatory effects of Eucommia ulmoides, Styphnolobium japonicum, and Portulaca oleracea extracts *via* TGF-β/Smad/IL-17 pathway. Chem. Biol. Technol. Agric. 12 (1), 139. 10.1186/s40538-025-00856-1

