# Supplementary material for: Vicenin-2 attenuates rosacea-like inflammation by inhibiting IL-17RA signaling
Source: Front Pharmacol. 2026 Mar 10;17:1793115. doi: 10.3389/fphar.2026.1793115 (PMC13010122; doi:10.3389/fphar.2026.1793115)

**Supplementary Materials**

**Part I. Mice**

| **Tabel S1. Raw erythema area measurements for animal model evaluation (cm2)** | | | | | | |
| --- | --- | --- | --- | --- | --- | --- |
| **Number** | **control** | **LL-37** | **LL-37+Vehicle** | **LL-37+0.025%VCN-2** | **LL-37+0.05%VCN-2** | **LL-37+0.1%VCN-2** |
| 1 | 0 | 1.39552 | 1.38578 | 1.28414 | 1.15223 | 0.93953 |
| 2 | 0 | 1.42587 | 1.35555 | 1.27186 | 1.22428 | 1.00092 |
| 3 | 0 | 1.37684 | 1.32348 | 1.2188 | 1.18299 | 0.82017 |
| 4 | 0 | 1.41055 | 1.49052 | 1.30637 | 1.14398 | 0.99148 |
| 5 | 0 | 1.35663 | 1.3719 | 1.31058 | 1.09842 | 0.80658 |
| Mean | 0 | 1.393 | 1.385 | 1.278 | 1.16 | 0.9117 |
| SEM | 0 | 0.0122 | 0.02825 | 0.0165 | 0.02094 | 0.04155 |
|  |  |  |  |  |  |  |
|  |  |  |  |  |  |  |
| **Tabel S2. Raw erythema score for animal model evaluation** | | | | | | |
| **Number** | **control** | **LL-37** | **LL-37+Vehicle** | **LL-37+0.025%VCN-2** | **LL-37+0.05%VCN-2** | **LL-37+0.1%VCN-2** |
| 1 | 0 | 4 | 4 | 3 | 2 | 2 |
| 2 | 0 | 3 | 4 | 2 | 1 | 2 |
| 3 | 0 | 3 | 4 | 3 | 3 | 1 |
| 4 | 0 | 4 | 3 | 2 | 1 | 1 |
| 5 | 0 | 3 | 4 | 3 | 2 | 0 |
| Mean | 0 | 3.4 | 3.8 | 2.6 | 1.8 | 1.2 |
| SEM | 0 | 0.2449 | 0.2 | 0.2449 | 0.3742 | 0.3742 |

**H&E staining of inflammatory cell count**

**Tabel S3. Raw Data for HE-Stained Inflammatory Cell Count**

| **Group** | **Number** | **Field of Review** | **Inflammatory cell count** | **Inflammatory cell count** | **Mean** | **SEM** |
| --- | --- | --- | --- | --- | --- | --- |
| **Control: A** | A1 | 400（1） | 0 | 0 | 0.00 | 0 |
|  |  | 400（2） | 0 |  |  |  |
|  |  | 400（3） | 0 |  |  |  |
|  | A2 | 400（1） | 0 | 0 |  |  |
|  |  | 400（2） | 0 |  |  |  |
|  |  | 400（3） | 0 |  |  |  |
|  | A3 | 400（1） | 0 | 0 |  |  |
|  |  | 400（2） | 0 |  |  |  |
|  |  | 400（3） | 0 |  |  |  |
| **LL-37: B** | B1 | 400（1） | 3 | 38 | 37.67 | 0.333 |
|  |  | 400（2） | 5 |  |  |  |
|  |  | 400（3） | 30 |  |  |  |
|  | B2 | 400（1） | 10 | 37 |  |  |
|  |  | 400（2） | 7 |  |  |  |
|  |  | 400（3） | 20 |  |  |  |
|  | B3 | 400（1） | 32 | 38 |  |  |
|  |  | 400（2） | 5 |  |  |  |
|  |  | 400（3） | 1 |  |  |  |
| **LL-37+Vehicle: C** | C1 | 400（1） | 18 | 37 | 37.00 | 1.155 |
|  |  | 400（2） | 6 |  |  |  |
|  |  | 400（3） | 13 |  |  |  |
|  | C2 | 400（1） | 9 | 39 |  |  |
|  |  | 400（2） | 15 |  |  |  |
|  |  | 400（3） | 15 |  |  |  |
|  | C3 | 400（1） | 16 | 35 |  |  |
|  |  | 400（2） | 16 |  |  |  |
|  |  | 400（3） | 3 |  |  |  |
| **LL-37+0.025%: D** | D1 | 400（1） | 9 | 23 | 25.00 | 1 |
|  |  | 400（2） | 4 |  |  |  |
|  |  | 400（3） | 10 |  |  |  |
|  | D2 | 400（1） | 11 | 26 |  |  |
|  |  | 400（2） | 10 |  |  |  |
|  |  | 400（3） | 5 |  |  |  |
|  | D3 | 400（1） | 7 | 26 |  |  |
|  |  | 400（2） | 9 |  |  |  |
|  |  | 400（3） | 10 |  |  |  |
| **LL-37+0.05%: E** | E1 | 400（1） | 9 | 15 | 15.67 | 0.667 |
|  |  | 400（2） | 4 |  |  |  |
|  |  | 400（3） | 2 |  |  |  |
|  | E2 | 400（1） | 7 | 17 |  |  |
|  |  | 400（2） | 5 |  |  |  |
|  |  | 400（3） | 5 |  |  |  |
|  | E3 | 400（1） | 1 | 15 |  |  |
|  |  | 400（2） | 6 |  |  |  |
|  |  | 400（3） | 8 |  |  |  |
| **LL-37+0.1%: F** | F1 | 400（1） | 4 | 13 | 12.00 | 1 |
|  |  | 400（2） | 4 |  |  |  |
|  |  | 400（3） | 5 |  |  |  |
|  | F2 | 400（1） | 2 | 10 |  |  |
|  |  | 400（2） | 4 |  |  |  |
|  |  | 400（3） | 4 |  |  |  |
|  | F3 | 400（1） | 5 | 13 |  |  |
|  |  | 400（2） | 4 |  |  |  |
|  |  | 400（3） | 4 |  |  |  |

**Toluidine blue staining of mast cell count**

| **Tabel S4. Raw Data for Toluidine Blue-Stained Mast Cell Counts** | | | | | | |
| --- | --- | --- | --- | --- | --- | --- |
| **Group** | **Number** | **Field of view** | **Number of mast cells** | **Number of mast cells** | **Mean** | **SEM** |
| **Control: A** | A1 | 400（1） | 1 | 5 | 4.67 | 0.88 |
|  |  | 400（2） | 3 |  |  |  |
|  |  | 400（3） | 1 |  |  |  |
|  | A2 | 400（1） | 2 | 6 |  |  |
|  |  | 400（2） | 3 |  |  |  |
|  |  | 400（3） | 1 |  |  |  |
|  | A3 | 400（1） | 1 | 3 |  |  |
|  |  | 400（2） | 1 |  |  |  |
|  |  | 400（3） | 1 |  |  |  |
| **LL-37: B** | B1 | 400（1） | 10 | 27 | 24.67 | 2.33 |
|  |  | 400（2） | 9 |  |  |  |
|  |  | 400（3） | 8 |  |  |  |
|  | B2 | 400（1） | 12 | 27 |  |  |
|  |  | 400（2） | 9 |  |  |  |
|  |  | 400（3） | 6 |  |  |  |
|  | B3 | 400（1） | 9 | 20 |  |  |
|  |  | 400（2） | 6 |  |  |  |
|  |  | 400（3） | 5 |  |  |  |
| **LL-37+Vehicle: C** | C1 | 400（1） | 9 | 24 | 22.33 | 0.88 |
|  |  | 400（2） | 8 |  |  |  |
|  |  | 400（3） | 7 |  |  |  |
|  | C2 | 400（1） | 8 | 22 |  |  |
|  |  | 400（2） | 5 |  |  |  |
|  |  | 400（3） | 9 |  |  |  |
|  | C3 | 400（1） | 5 | 21 |  |  |
|  |  | 400（2） | 7 |  |  |  |
|  |  | 400（3） | 9 |  |  |  |
| **LL-37+0.025%: D** | D1 | 400（1） | 4 | 20 | 18.33 | 1.67 |
|  |  | 400（2） | 6 |  |  |  |
|  |  | 400（3） | 10 |  |  |  |
|  | D2 | 400（1） | 5 | 15 |  |  |
|  |  | 400（2） | 4 |  |  |  |
|  |  | 400（3） | 6 |  |  |  |
|  | D3 | 400（1） | 7 | 20 |  |  |
|  |  | 400（2） | 9 |  |  |  |
|  |  | 400（3） | 4 |  |  |  |
| **LL-37+0.05%: E** | E1 | 400（1） | 6 | 13 | 12.33 | 0.67 |
|  |  | 400（2） | 3 |  |  |  |
|  |  | 400（3） | 4 |  |  |  |
|  | E2 | 400（1） | 4 | 11 |  |  |
|  |  | 400（2） | 3 |  |  |  |
|  |  | 400（3） | 4 |  |  |  |
|  | E3 | 400（1） | 6 | 13 |  |  |
|  |  | 400（2） | 4 |  |  |  |
|  |  | 400（3） | 3 |  |  |  |
| **LL-37+0.1%: F** | F1 | 400（1） | 2 | 6 | 6.00 | 1.16 |
|  |  | 400（2） | 1 |  |  |  |
|  |  | 400（3） | 3 |  |  |  |
|  | F2 | 400（1） | 1 | 4 |  |  |
|  |  | 400（2） | 3 |  |  |  |
|  |  | 400（3） | 0 |  |  |  |
|  | F3 | 400（1） | 4 | 8 |  |  |
|  |  | 400（2） | 2 |  |  |  |
|  |  | 400（3） | 2 |  |  |  |

**Immunofluorescence**

| **Tabel S5. Raw data of** **immunofluorescence staining** | | | | | | | | |  |
| --- | --- | --- | --- | --- | --- | --- | --- | --- | --- |
| **Sample Grouping** | **Number** | **400× Image ID** | **area（sum）** | **Integrated Optical Density（IOD）** | **Density (mean）** | **Density (mean）** | **Density (mean）** | **SEM** |  |
|  |  |  |  |  |  |  |  |  |  |
| 1.Control: A | A1 | 1-7 | 1920000 | 24.64 | 0.00001 | 0.00004 | 0.00013 | 0.00006 |  |
|  |  | 1-9 | 1920000 | 177.77 | 0.00009 |  |  |  |  |
|  |  | 1-11 | 1920000 | 8.07 | 0.00000 |  |  |  |  |
|  | A2 | 1-7 | 1920000 | 436.50 | 0.00023 | 0.00011 |  |  |  |
|  |  | 1-9 | 1920000 | 91.19 | 0.00005 |  |  |  |  |
|  |  | 1-11 | 1920000 | 79.82 | 0.00004 |  |  |  |  |
|  | A3 | 1-7 | 1920000 | 123.25 | 0.00006 | 0.00024 |  |  |  |
|  |  | 1-9 | 1920000 | 422.54 | 0.00022 |  |  |  |  |
|  |  | 1-11 | 1920000 | 851.06 | 0.00044 |  |  |  |  |
| 2.LL-37: B | B1 | 1-7 | 1920000 | 8645.56 | 0.00450 | 0.00355 | 0.00319 | 0.00022 |  |
|  |  | 1-9 | 1920000 | 4202.71 | 0.00219 |  |  |  |  |
|  |  | 1-11 | 1920000 | 7621.75 | 0.00397 |  |  |  |  |
|  | B2 | 1-7 | 1920000 | 7510.36 | 0.00391 | 0.00321 |  |  |  |
|  |  | 1-9 | 1920000 | 6921.62 | 0.00361 |  |  |  |  |
|  |  | 1-11 | 1920000 | 4059.40 | 0.00211 |  |  |  |  |
|  | B3 | 1-7 | 1920000 | 5254.59 | 0.00274 | 0.00280 |  |  |  |
|  |  | 1-9 | 1920000 | 4170.43 | 0.00217 |  |  |  |  |
|  |  | 1-11 | 1920000 | 6731.02 | 0.00351 |  |  |  |  |
| 3.LL-37+Vehicle: C | C1 | 1-7 | 1920000 | 9212.46 | 0.00480 | 0.00411 | 0.00372 | 0.00028 |  |
|  |  | 1-9 | 1920000 | 6068.64 | 0.00316 |  |  |  |  |
|  |  | 1-11 | 1920000 | 8381.59 | 0.00437 |  |  |  |  |
|  | C2 | 1-7 | 1920000 | 9775.95 | 0.00509 | 0.00387 |  |  |  |
|  |  | 1-9 | 1920000 | 7819.43 | 0.00407 |  |  |  |  |
|  |  | 1-11 | 1920000 | 4668.36 | 0.00243 |  |  |  |  |
|  | C3 | 1-7 | 1920000 | 9166.04 | 0.00477 | 0.00319 |  |  |  |
|  |  | 1-9 | 1920000 | 5382.82 | 0.00280 |  |  |  |  |
|  |  | 1-11 | 1920000 | 3800.58 | 0.00198 |  |  |  |  |
| 4.LL-37+0.025%VCN-2: D | D1 | 1-7 | 1920000 | 3562.62 | 0.00186 | 0.00172 | 0.00146 | 0.00025 |  |
|  |  | 1-9 | 1920000 | 3210.41 | 0.00167 |  |  |  |  |
|  |  | 1-11 | 1920000 | 3160.48 | 0.00165 |  |  |  |  |
|  | D2 | 1-7 | 1920000 | 3566.95 | 0.00186 | 0.00170 |  |  |  |
|  |  | 1-9 | 1920000 | 2312.59 | 0.00120 |  |  |  |  |
|  |  | 1-11 | 1920000 | 3935.82 | 0.00205 |  |  |  |  |
|  | D3 | 1-7 | 1920000 | 1438.41 | 0.00075 | 0.00095 |  |  |  |
|  |  | 1-9 | 1920000 | 2502.94 | 0.00130 |  |  |  |  |
|  |  | 1-11 | 1920000 | 1506.09 | 0.00078 |  |  |  |  |
| 5.LL-37+0.05%VCN-2: E | E1 | 1-7 | 1920000 | 2333.18 | 0.00122 | 0.00136 | 0.00111 | 0.00012 |  |
|  |  | 1-9 | 1920000 | 1919.72 | 0.00100 |  |  |  |  |
|  |  | 1-11 | 1920000 | 3563.15 | 0.00186 |  |  |  |  |
|  | E2 | 1-7 | 1920000 | 2056.65 | 0.00107 | 0.00102 |  |  |  |
|  |  | 1-9 | 1920000 | 2247.48 | 0.00117 |  |  |  |  |
|  |  | 1-11 | 1920000 | 1547.68 | 0.00081 |  |  |  |  |
|  | E3 | 1-7 | 1920000 | 1322.69 | 0.00069 | 0.00097 |  |  |  |
|  |  | 1-9 | 1920000 | 2705.05 | 0.00141 |  |  |  |  |
|  |  | 1-11 | 1920000 | 1545.17 | 0.00080 |  |  |  |  |
| 6.LL-37+0.1%VCN-2: F | F1 | 1-7 | 1920000 | 451.16 | 0.00023 | 0.00043 | 0.00054 | 0.00005 |  |
|  |  | 1-9 | 1920000 | 855.95 | 0.00045 |  |  |  |  |
|  |  | 1-11 | 1920000 | 1186.46 | 0.00062 |  |  |  |  |
|  | F2 | 1-7 | 1920000 | 1488.96 | 0.00078 | 0.00060 |  |  |  |
|  |  | 1-9 | 1920000 | 791.85 | 0.00041 |  |  |  |  |
|  |  | 1-11 | 1920000 | 1153.85 | 0.00060 |  |  |  |  |
|  | F3 | 1-7 | 1920000 | 1413.31 | 0.00074 | 0.00058 |  |  |  |
|  |  | 1-9 | 1920000 | 1176.37 | 0.00061 |  |  |  |  |
|  |  | 1-11 | 1920000 | 763.90 | 0.00040 |  |  |  |  |

**Immunohistochemical staining of p-p65**

| **Tabel S6. Raw Data from** **p-p65 Immunohistochemistry in Mice** | | | | | | | | |  |
| --- | --- | --- | --- | --- | --- | --- | --- | --- | --- |
| **Sample Grouping** | **Number** | **400× Image ID** | **area（sum）** | **Integrated Optical Density（IOD）** | **Density (mean）** | **Density (mean）** | **Density (mean）** | **SEM** |  |
|  |  |  |  |  |  |  |  |  |  |
| 1.Control: A | A1 | 400（1） | 1920000 | 3951.5847 | 0.00206 | 0.00259 | 0.00210 | 0.00043 |  |
|  |  | 400（2） | 1920000 | 6149.1592 | 0.00320 |  |  |  |  |
|  |  | 400（3） | 1920000 | 4758.8505 | 0.00248 |  |  |  |  |
|  |  | 400（4） | 1920000 | 5033.7216 | 0.00262 |  |  |  |  |
|  | A2 | 400（1） | 1920000 | 2100.3672 | 0.00109 | 0.00245 |  |  |  |
|  |  | 400（2） | 1920000 | 8503.6347 | 0.00443 |  |  |  |  |
|  |  | 400（3） | 1920000 | 4957.6245 | 0.00258 |  |  |  |  |
|  |  | 400（4） | 1920000 | 3251.2698 | 0.00169 |  |  |  |  |
|  | A3 | 400（1） | 1920000 | 1382.5631 | 0.00072 | 0.00125 |  |  |  |
|  |  | 400（2） | 1920000 | 967.9619 | 0.00050 |  |  |  |  |
|  |  | 400（3） | 1920000 | 3352.8247 | 0.00175 |  |  |  |  |
|  |  | 400（4） | 1920000 | 3885.7559 | 0.00202 |  |  |  |  |
| 2.LL-37: B | B1 | 400（1） | 1920000 | 32874.3320 | 0.01712 | 0.01043 | 0.01110 | 0.00063 |  |
|  |  | 400（2） | 1920000 | 19906.4355 | 0.01037 |  |  |  |  |
|  |  | 400（3） | 1920000 | 14263.9932 | 0.00743 |  |  |  |  |
|  |  | 400（4） | 1920000 | 13085.1064 | 0.00682 |  |  |  |  |
|  | B2 | 400（1） | 1920000 | 16840.9971 | 0.00877 | 0.01052 |  |  |  |
|  |  | 400（2） | 1920000 | 15185.0098 | 0.00791 |  |  |  |  |
|  |  | 400（3） | 1920000 | 28027.5117 | 0.01460 |  |  |  |  |
|  |  | 400（4） | 1920000 | 20751.6211 | 0.01081 |  |  |  |  |
|  | B3 | 400（1） | 1920000 | 17671.1436 | 0.00920 | 0.01235 |  |  |  |
|  |  | 400（2） | 1920000 | 12691.1533 | 0.00661 |  |  |  |  |
|  |  | 400（3） | 1920000 | 27729.1211 | 0.01444 |  |  |  |  |
|  |  | 400（4） | 1920000 | 36750.0391 | 0.01914 |  |  |  |  |
| 3.LL-37+Vehicle: C | C1 | 400（1） | 1920000 | 14128.0068 | 0.00736 | 0.00755 | 0.01128 | 0.00203 |  |
|  |  | 400（2） | 1920000 | 25579.2793 | 0.01332 |  |  |  |  |
|  |  | 400（3） | 1920000 | 13320.5800 | 0.00694 |  |  |  |  |
|  |  | 400（4） | 1920000 | 4951.8276 | 0.00258 |  |  |  |  |
|  | C2 | 400（1） | 1920000 | 22574.8339 | 0.01176 | 0.01179 |  |  |  |
|  |  | 400（2） | 1920000 | 19602.3594 | 0.01021 |  |  |  |  |
|  |  | 400（3） | 1920000 | 21259.6523 | 0.01107 |  |  |  |  |
|  |  | 400（4） | 1920000 | 27091.2957 | 0.01411 |  |  |  |  |
|  | C3 | 400（1） | 1920000 | 21781.3145 | 0.01134 | 0.01452 |  |  |  |
|  |  | 400（2） | 1920000 | 33932.1133 | 0.01767 |  |  |  |  |
|  |  | 400（3） | 1920000 | 30173.8027 | 0.01572 |  |  |  |  |
|  |  | 400（4） | 1920000 | 25594.5371 | 0.01333 |  |  |  |  |
| 4.LL-37+0.025%VCN-2: D | D1 | 400（1） | 1920000 | 10536.0498 | 0.00549 | 0.00620 | 0.00793 | 0.00087 |  |
|  |  | 400（2） | 1920000 | 11518.8311 | 0.00600 |  |  |  |  |
|  |  | 400（3） | 1920000 | 11752.8828 | 0.00612 |  |  |  |  |
|  |  | 400（4） | 1920000 | 13789.1582 | 0.00718 |  |  |  |  |
|  | D2 | 400（1） | 1920000 | 9920.5009 | 0.00517 | 0.00861 |  |  |  |
|  |  | 400（2） | 1920000 | 10519.0234 | 0.00548 |  |  |  |  |
|  |  | 400（3） | 1920000 | 19933.7285 | 0.01038 |  |  |  |  |
|  |  | 400（4） | 1920000 | 25756.4238 | 0.01341 |  |  |  |  |
|  | D3 | 400（1） | 1920000 | 15199.3369 | 0.00792 | 0.00897 |  |  |  |
|  |  | 400（2） | 1920000 | 11225.8330 | 0.00585 |  |  |  |  |
|  |  | 400（3） | 1920000 | 24532.8105 | 0.01278 |  |  |  |  |
|  |  | 400（4） | 1920000 | 17927.4941 | 0.00934 |  |  |  |  |
| 5.LL-37+0.05%VCN-2: E | E1 | 400（1） | 1920000 | 14576.5996 | 0.00759 | 0.00756 | 0.00673 | 0.00082 |  |
|  |  | 400（2） | 1920000 | 20359.1836 | 0.01060 |  |  |  |  |
|  |  | 400（3） | 1920000 | 11403.2598 | 0.00594 |  |  |  |  |
|  |  | 400（4） | 1920000 | 11745.6758 | 0.00612 |  |  |  |  |
|  | E2 | 400（1） | 1920000 | 13383.8896 | 0.00697 | 0.00509 |  |  |  |
|  |  | 400（2） | 1920000 | 9972.4937 | 0.00519 |  |  |  |  |
|  |  | 400（3） | 1920000 | 7220.5320 | 0.00376 |  |  |  |  |
|  |  | 400（4） | 1920000 | 8531.2725 | 0.00444 |  |  |  |  |
|  | E3 | 400（1） | 1920000 | 11750.0322 | 0.00612 | 0.00753 |  |  |  |
|  |  | 400（2） | 1920000 | 14486.6807 | 0.00755 |  |  |  |  |
|  |  | 400（3） | 1920000 | 14518.4727 | 0.00756 |  |  |  |  |
|  |  | 400（4） | 1920000 | 17092.5605 | 0.00890 |  |  |  |  |
| 6.LL-37+0.1%VCN-2: F | F1 | 400（1） | 1920000 | 3855.9976 | 0.00201 | 0.00395 | 0.00358 | 0.00027 |  |
|  |  | 400（2） | 1920000 | 6012.7378 | 0.00313 |  |  |  |  |
|  |  | 400（3） | 1920000 | 9519.0010 | 0.00496 |  |  |  |  |
|  |  | 400（4） | 1920000 | 10956.2080 | 0.00571 |  |  |  |  |
|  | F2 | 400（1） | 1920000 | 6612.3501 | 0.00344 | 0.00375 |  |  |  |
|  |  | 400（2） | 1920000 | 6747.7603 | 0.00351 |  |  |  |  |
|  |  | 400（3） | 1920000 | 5879.0463 | 0.00306 |  |  |  |  |
|  |  | 400（4） | 1920000 | 9539.1836 | 0.00497 |  |  |  |  |
|  | F3 | 400（1） | 1920000 | 3133.2827 | 0.00163 | 0.00305 |  |  |  |
|  |  | 400（2） | 1920000 | 9040.8212 | 0.00471 |  |  |  |  |
|  |  | 400（3） | 1920000 | 6343.7177 | 0.00330 |  |  |  |  |
|  |  | 400（4） | 1920000 | 4904.6045 | 0.00255 |  |  |  |  |

**Mouse ELISA experiment**

| **Tabel S7.** Mouse ELISA Raw Data of TNF-α | | | | | |
| --- | --- | --- | --- | --- | --- |
| Sample | OD Value | Mean minus blank OD value | Concentration of TNF-α(pg/ml) | AVERAGE | SEM |
| Control | 0.321 | 0.223 | 71.424 | 83.12 | 6.58 |
|  | 0.378 | 0.280 | 94.192 |  |  |
|  | 0.352 | 0.254 | 83.737 |  |  |
| LL-37 | 0.846 | 0.748 | 305.871 | 332.4 | 15.87 |
|  | 0.951 | 0.853 | 360.742 |  |  |
|  | 0.894 | 0.796 | 330.561 |  |  |
| LL-37+Vehicle | 0.987 | 0.889 | 380.309 | 346.7 | 19.58 |
|  | 0.859 | 0.761 | 312.495 |  |  |
|  | 0.926 | 0.828 | 347.387 |  |  |
| LL-37+0.025%vicenin-2 | 0.737 | 0.639 | 252.099 | 272.6 | 11.6 |
|  | 0.781 | 0.683 | 273.436 |  |  |
|  | 0.819 | 0.721 | 292.262 |  |  |
| LL-37+0.025%vicenin-2 | 0.688 | 0.590 | 228.897 | 227.9 | 10.66 |
|  | 0.645 | 0.547 | 209.002 |  |  |
|  | 0.724 | 0.626 | 245.887 |  |  |
| LL-37+0.025%vicenin-2 | 0.453 | 0.355 | 125.029 | 124.7 | 6.878 |
|  | 0.480 | 0.382 | 136.387 |  |  |
|  | 0.423 | 0.325 | 112.570 |  |  |
|  |  |  |  |  |  |
|  |  |  |  |  |  |
| **Tabel S8.** Mouse ELISA Raw Data of IL-6 | | | | | |
| Sample | OD Value | Mean minus blank OD value | Concentration of TNF-α(pg/ml) | AVERAGE | SEM |
| Control | 0.118 | 0.036 | 111.200 | 112.7 | 8.191 |
|  | 0.125 | 0.043 | 127.617 |  |  |
|  | 0.113 | 0.031 | 99.368 |  |  |
| LL-37 | 0.284 | 0.202 | 463.680 | 425.6 | 23.51 |
|  | 0.243 | 0.161 | 382.693 |  |  |
|  | 0.267 | 0.185 | 430.488 |  |  |
| LL-37+Vehicle | 0.231 | 0.149 | 358.352 | 395.2 | 20.84 |
|  | 0.250 | 0.168 | 396.752 |  |  |
|  | 0.267 | 0.185 | 430.488 |  |  |
| LL-37+0.025%vicenin-2 | 0.201 | 0.119 | 296.081 | 305.7 | 17.61 |
|  | 0.194 | 0.112 | 281.239 |  |  |
|  | 0.222 | 0.140 | 339.890 |  |  |
| LL-37+0.025%vicenin-2 | 0.169 | 0.087 | 227.183 | 248.2 | 11.38 |
|  | 0.180 | 0.098 | 251.175 |  |  |
|  | 0.187 | 0.105 | 266.272 |  |  |
| LL-37+0.025%vicenin-2 | 0.146 | 0.064 | 175.888 | 192.3 | 10.45 |
|  | 0.162 | 0.080 | 211.737 |  |  |
|  | 0.152 | 0.070 | 189.422 |  |  |
|  |  |  |  |  |  |
|  |  |  |  |  |  |
| **Tabel S9.** Mouse ELISA Raw Data of IL-1β | | | | | |
| Sample | OD Value | Mean minus blank OD value | Concentration of TNF-α(pg/ml) | AVERAGE | SEM |
| Control | 0.231 | 0.126 | 142.678 | 130 | 8.243 |
|  | 0.208 | 0.103 | 114.557 |  |  |
|  | 0.223 | 0.118 | 132.902 |  |  |
| LL-37 | 0.417 | 0.312 | 369.772 | 403.8 | 20.26 |
|  | 0.443 | 0.338 | 401.683 |  |  |
|  | 0.474 | 0.369 | 439.856 |  |  |
| LL-37+Vehicle | 0.397 | 0.292 | 345.278 | 382.1 | 21.62 |
|  | 0.458 | 0.353 | 420.136 |  |  |
|  | 0.426 | 0.321 | 380.809 |  |  |
| LL-37+0.025%vicenin-2 | 0.382 | 0.277 | 326.932 | 324.9 | 18.02 |
|  | 0.354 | 0.249 | 292.732 |  |  |
|  | 0.405 | 0.300 | 355.070 |  |  |
| LL-37+0.025%vicenin-2 | 0.327 | 0.222 | 259.791 | 235.4 | 13.43 |
|  | 0.289 | 0.184 | 213.455 |  |  |
|  | 0.305 | 0.200 | 232.964 |  |  |
| LL-37+0.025%vicenin-2 | 0.269 | 0.164 | 189.064 | 185.4 | 8.305 |
|  | 0.253 | 0.148 | 169.542 |  |  |
|  | 0.276 | 0.171 | 197.602 |  |  |
|  |  |  |  |  |  |
|  |  |  |  |  |  |
| Mouse ELISA Raw Data of CXCL1 | | | | | |
| Sample | OD Value | Mean minus blank OD value | Concentration of TNF-α(pg/ml) | AVERAGE | SEM |
| Control | 0.158 | 0.051 | 89.453 | 93.41 | 5.511 |
|  | 0.156 | 0.049 | 86.479 |  |  |
|  | 0.168 | 0.061 | 104.297 |  |  |
| LL-37 | 0.325 | 0.218 | 332.744 | 300.6 | 17.54 |
|  | 0.300 | 0.193 | 296.835 |  |  |
|  | 0.283 | 0.176 | 272.334 |  |  |
| LL-37+Vehicle | 0.267 | 0.160 | 249.208 | 280 | 17.49 |
|  | 0.309 | 0.202 | 309.778 |  |  |
|  | 0.289 | 0.182 | 280.990 |  |  |
| LL-37+0.025%vicenin-2 | 0.254 | 0.147 | 230.365 | 234.7 | 10.69 |
|  | 0.271 | 0.164 | 254.996 |  |  |
|  | 0.246 | 0.139 | 218.745 |  |  |
| LL-37+0.025%vicenin-2 | 0.221 | 0.114 | 182.301 | 183.7 | 8.889 |
|  | 0.212 | 0.105 | 169.129 |  |  |
|  | 0.233 | 0.126 | 199.819 |  |  |
| LL-37+0.025%vicenin-2 | 0.192 | 0.085 | 139.754 | 139.3 | 7.232 |
|  | 0.200 | 0.093 | 151.522 |  |  |
|  | 0.183 | 0.076 | 126.485 |  |  |

**Mouse RT-qPCR experiment**

| **Tabel S10. Raw Data of RT-qPCR** | | | | | | | | | | | | | | | |  |  |  |
| --- | --- | --- | --- | --- | --- | --- | --- | --- | --- | --- | --- | --- | --- | --- | --- | --- | --- | --- |
|  | **Control** | | | **LL-37** | | | **LL-37+Vehicle** | | | **LL-37+0.025%vicenin-2** | | | **LL-37+0.05%vicenin-2** | | | **LL-37+0.1%vicenin-2** | | |
| **Mus GAPDH** | 18.90 | 18.71 | 18.61 | 18.53 | 18.73 | 18.83 | 17.68 | 18.30 | 17.53 | 18.76 | 18.62 | 18.21 | 17.66 | 17.86 | 18.20 | 17.99 | 17.88 | 18.19 |
|  | 18.82 | 18.57 | 18.85 | 18.41 | 18.78 | 18.97 | 17.90 | 18.08 | 17.63 | 19.13 | 18.67 | 17.90 | 17.72 | 17.74 | 18.41 | 17.54 | 17.52 | 18.46 |
|  | 18.86 | 18.87 | 18.99 | 18.36 | 19.00 | 18.74 | 17.82 | 18.36 | 17.92 | 18.80 | 18.55 | 18.08 | 17.68 | 17.62 | 18.44 | 17.77 | 17.93 | 18.49 |
|  |  |  |  |  |  |  |  |  |  |  |  |  |  |  |  |  |  |  |
|  |  |  |  |  |  |  |  |  |  |  |  |  |  |  |  |  |  |  |
|  | **Control** | | | **LL-37** | | | **LL-37+Vehicle** | | | **LL-37+0.025%vicenin-2** | | | **LL-37+0.05%vicenin-2** | | | **LL-37+0.1%vicenin-2** | | |
| **Mus IL-6** | 31.40 | 30.99 | 31.75 | 28.95 | 29.63 | 29.47 | 28.44 | 28.65 | 28.35 | 30.12 | 29.71 | 28.98 | 28.95 | 29.30 | 29.58 | 29.61 | 29.60 | 30.14 |
|  | 31.20 | 30.76 | 31.56 | 28.94 | 29.39 | 29.28 | 28.69 | 28.87 | 28.54 | 30.16 | 29.80 | 29.26 | 29.14 | 29.03 | 29.50 | 29.50 | 29.28 | 30.02 |
|  | 31.55 | 30.95 | 31.50 | 29.37 | 29.24 | 29.42 | 28.41 | 28.90 | 28.71 | 29.77 | 29.93 | 29.13 | 29.08 | 29.22 | 29.64 | 29.75 | 29.35 | 29.99 |
|  |  |  |  |  |  |  |  |  |  |  |  |  |  |  |  |  |  |  |
| Δt | 12.50 | 12.28 | 13.14 | 10.42 | 10.89 | 10.64 | 10.76 | 10.35 | 10.82 | 11.36 | 11.09 | 10.76 | 11.29 | 11.44 | 11.38 | 11.62 | 11.72 | 11.95 |
|  | 12.38 | 12.19 | 12.71 | 10.53 | 10.61 | 10.31 | 10.79 | 10.79 | 10.91 | 11.03 | 11.13 | 11.35 | 11.42 | 11.29 | 11.09 | 11.96 | 11.76 | 11.56 |
|  | 12.69 | 12.08 | 12.51 | 11.01 | 10.24 | 10.69 | 10.59 | 10.54 | 10.79 | 10.97 | 11.38 | 11.06 | 11.39 | 11.60 | 11.20 | 11.97 | 11.41 | 11.49 |
|  |  |  |  |  |  |  |  |  |  |  |  |  |  |  |  |  |  |  |
| ΔΔt | 0.00 | -0.22 | 0.64 | -2.08 | -1.61 | -1.87 | -1.74 | -2.15 | -1.68 | -1.14 | -1.41 | -1.74 | -1.21 | -1.06 | -1.12 | -0.88 | -0.78 | -0.55 |
|  | 0.00 | -0.19 | 0.33 | -1.85 | -1.77 | -2.06 | -1.59 | -1.59 | -1.47 | -1.35 | -1.24 | -1.02 | -0.96 | -1.09 | -1.29 | -0.42 | -0.62 | -0.82 |
|  | 0.00 | -0.60 | -0.18 | -1.68 | -2.45 | -2.00 | -2.10 | -2.14 | -1.90 | -1.72 | -1.31 | -1.63 | -1.29 | -1.09 | -1.48 | -0.71 | -1.27 | -1.19 |
|  |  |  |  |  |  |  |  |  |  |  |  |  |  |  |  |  |  |  |
| 2-ΔΔt | 1.00 | 1.17 | 0.64 | 4.23 | 3.05 | 3.65 | 3.35 | 4.44 | 3.20 | 2.21 | 2.65 | 3.34 | 2.31 | 2.08 | 2.17 | 1.84 | 1.72 | 1.46 |
|  | 1.00 | 1.14 | 0.80 | 3.60 | 3.41 | 4.18 | 3.00 | 3.01 | 2.77 | 2.55 | 2.37 | 2.03 | 1.94 | 2.13 | 2.44 | 1.33 | 1.54 | 1.77 |
|  | 1.00 | 1.52 | 1.13 | 3.20 | 5.45 | 4.00 | 4.28 | 4.42 | 3.73 | 3.29 | 2.47 | 3.10 | 2.45 | 2.12 | 2.80 | 1.64 | 2.42 | 2.28 |
| Mean | 1.04 | | | 3.86 | | | 3.58 | | | 2.67 | | | 2.27 | | | 1.78 | | |
| SEM | 0.12 | | | 0.09 | | | 0.21 | | | 0.09 | | | 0.11 | | | 0.09 | | |
|  |  |  |  |  |  |  |  |  |  |  |  |  |  |  |  |  |  |  |
|  |  |  |  |  |  |  |  |  |  |  |  |  |  |  |  |  |  |  |
|  | **Control** | | | **LL-37** | | | **LL-37+Vehicle** | | | **LL-37+0.025%vicenin-2** | | | **LL-37+0.05%vicenin-2** | | | **LL-37+0.1%vicenin-2** | | |
| **Mus GAPDH** | 19.14 | 18.59 | 18.68 | 18.34 | 17.72 | 18.89 | 18.44 | 18.97 | 19.18 | 17.81 | 18.35 | 18.53 | 17.75 | 18.57 | 18.26 | 19.35 | 19.25 | 19.32 |
|  | 19.37 | 18.50 | 18.43 | 17.88 | 17.56 | 18.74 | 17.95 | 18.53 | 19.40 | 17.51 | 18.46 | 18.78 | 17.58 | 18.97 | 18.16 | 19.05 | 18.86 | 19.08 |
|  | 19.07 | 18.36 | 18.48 | 18.10 | 17.83 | 18.44 | 18.05 | 18.85 | 19.29 | 17.66 | 18.47 | 18.43 | 17.67 | 18.84 | 18.31 | 19.22 | 19.22 | 18.92 |
|  |  |  |  |  |  |  |  |  |  |  |  |  |  |  |  |  |  |  |
|  |  |  |  |  |  |  |  |  |  |  |  |  |  |  |  |  |  |  |
|  | **Control** | | | **LL-37** | | | **LL-37+Vehicle** | | | **LL-37+0.025%vicenin-2** | | | **LL-37+0.05%vicenin-2** | | | **LL-37+0.1%vicenin-2** | | |
| **Mus TNF-α** | 32.31 | 32.23 | 31.79 | 29.37 | 29.33 | 30.07 | 29.57 | 30.35 | 30.59 | 28.97 | 29.91 | 30.40 | 29.75 | 30.53 | 29.81 | 32.27 | 31.81 | 31.81 |
|  | 32.57 | 32.01 | 31.61 | 29.58 | 28.92 | 29.77 | 29.32 | 30.25 | 30.62 | 29.15 | 30.21 | 30.55 | 29.83 | 30.75 | 29.97 | 31.98 | 31.53 | 31.91 |
|  | 32.74 | 31.91 | 31.91 | 29.73 | 29.06 | 29.81 | 29.40 | 30.10 | 30.26 | 29.35 | 30.00 | 30.20 | 29.62 | 30.89 | 30.28 | 31.80 | 31.76 | 32.28 |
|  |  |  |  |  |  |  |  |  |  |  |  |  |  |  |  |  |  |  |
| Δt | 13.17 | 13.64 | 13.12 | 11.03 | 11.61 | 11.17 | 11.13 | 11.38 | 11.40 | 11.15 | 11.56 | 11.87 | 12.00 | 11.95 | 11.55 | 12.91 | 12.56 | 12.48 |
|  | 13.20 | 13.51 | 13.18 | 11.71 | 11.36 | 11.04 | 11.37 | 11.72 | 11.22 | 11.64 | 11.74 | 11.78 | 12.26 | 11.78 | 11.81 | 12.92 | 12.66 | 12.83 |
|  | 13.66 | 13.55 | 13.43 | 11.63 | 11.23 | 11.36 | 11.35 | 11.24 | 10.97 | 11.69 | 11.53 | 11.77 | 11.96 | 12.05 | 11.97 | 12.58 | 12.53 | 13.37 |
|  |  |  |  |  |  |  |  |  |  |  |  |  |  |  |  |  |  |  |
| ΔΔt | 0.00 | 0.48 | -0.05 | -2.14 | -1.56 | -1.99 | -2.03 | -1.78 | -1.76 | -2.01 | -1.61 | -1.29 | -1.16 | -1.21 | -1.61 | -0.26 | -0.60 | -0.68 |
|  | 0.00 | 0.31 | -0.02 | -1.50 | -1.84 | -2.16 | -1.83 | -1.48 | -1.98 | -1.56 | -1.46 | -1.43 | -0.94 | -1.42 | -1.39 | -0.28 | -0.54 | -0.37 |
|  | 0.00 | -0.11 | -0.23 | -2.03 | -2.43 | -2.30 | -2.31 | -2.42 | -2.69 | -1.97 | -2.14 | -1.89 | -1.71 | -1.62 | -1.69 | -1.08 | -1.13 | -0.29 |
|  |  |  |  |  |  |  |  |  |  |  |  |  |  |  |  |  |  |  |
| 2-ΔΔt | 1.00 | 0.72 | 1.04 | 4.41 | 2.95 | 3.98 | 4.09 | 3.44 | 3.39 | 4.03 | 3.05 | 2.45 | 2.24 | 2.32 | 3.06 | 1.19 | 1.52 | 1.61 |
|  | 1.00 | 0.81 | 1.02 | 2.82 | 3.59 | 4.48 | 3.55 | 2.79 | 3.96 | 2.95 | 2.75 | 2.69 | 1.92 | 2.68 | 2.62 | 1.21 | 1.45 | 1.29 |
|  | 1.00 | 1.08 | 1.18 | 4.09 | 5.38 | 4.92 | 4.95 | 5.35 | 6.46 | 3.92 | 4.40 | 3.71 | 3.26 | 3.06 | 3.23 | 2.12 | 2.19 | 1.23 |
| Mean | 0.98 | | | 4.07 | | | 4.22 | | | 3.33 | | | 2.71 | | | 1.53 | | |
| SEM | 0.06 | | | 0.20 | | | 0.22 | | | 0.20 | | | 0.14 | | | 0.10 | | |
|  |  |  |  |  |  |  |  |  |  |  |  |  |  |  |  |  |  |  |
|  |  |  |  |  |  |  |  |  |  |  |  |  |  |  |  |  |  |  |
|  | **Control** | | | **LL-37** | | | **LL-37+Vehicle** | | | **LL-37+0.025%vicenin-2** | | | **LL-37+0.05%vicenin-2** | | | **LL-37+0.1%vicenin-2** | | |
| **Mus IL-1β** | 29.00 | 27.97 | 28.69 | 26.35 | 25.80 | 26.59 | 26.46 | 26.99 | 27.71 | 26.22 | 27.05 | 27.16 | 26.64 | 27.84 | 27.22 | 28.46 | 28.61 | 28.34 |
|  | 29.04 | 28.23 | 28.44 | 26.44 | 25.97 | 26.93 | 26.58 | 27.31 | 27.44 | 26.12 | 26.73 | 27.27 | 26.87 | 27.74 | 27.12 | 28.62 | 28.54 | 28.51 |
|  | 29.14 | 28.14 | 28.30 | 26.64 | 26.03 | 26.86 | 26.74 | 27.18 | 27.38 | 26.45 | 26.84 | 27.35 | 26.94 | 27.50 | 27.39 | 28.33 | 28.78 | 28.65 |
|  |  |  |  |  |  |  |  |  |  |  |  |  |  |  |  |  |  |  |
| Δt | 9.86 | 9.39 | 10.01 | 8.01 | 8.07 | 7.70 | 8.02 | 8.02 | 8.52 | 8.41 | 8.70 | 8.63 | 8.89 | 9.27 | 8.96 | 9.11 | 9.36 | 9.01 |
|  | 9.67 | 9.73 | 10.01 | 8.56 | 8.41 | 8.19 | 8.63 | 8.78 | 8.04 | 8.61 | 8.27 | 8.50 | 9.30 | 8.77 | 8.96 | 9.56 | 9.67 | 9.44 |
|  | 10.07 | 9.78 | 9.82 | 8.54 | 8.20 | 8.41 | 8.69 | 8.33 | 8.09 | 8.79 | 8.36 | 8.92 | 9.28 | 8.66 | 9.08 | 9.11 | 9.55 | 9.74 |
|  |  |  |  |  |  |  |  |  |  |  |  |  |  |  |  |  |  |  |
| ΔΔt | 0.00 | -0.47 | 0.15 | -1.85 | -1.78 | -2.16 | -1.83 | -1.84 | -1.34 | -1.45 | -1.15 | -1.23 | -0.97 | -0.59 | -0.90 | -0.75 | -0.50 | -0.84 |
|  | 0.00 | 0.07 | 0.34 | -1.11 | -1.26 | -1.48 | -1.04 | -0.89 | -1.63 | -1.06 | -1.40 | -1.17 | -0.37 | -0.90 | -0.71 | -0.10 | 0.01 | -0.23 |
|  | 0.00 | -0.29 | -0.24 | -1.52 | -1.86 | -1.65 | -1.37 | -1.74 | -1.98 | -1.28 | -1.70 | -1.14 | -0.79 | -1.40 | -0.99 | -0.96 | -0.51 | -0.33 |
|  |  |  |  |  |  |  |  |  |  |  |  |  |  |  |  |  |  |  |
| 2-ΔΔt | 1.00 | 1.39 | 0.90 | 3.60 | 3.44 | 4.46 | 3.56 | 3.58 | 2.52 | 2.73 | 2.22 | 2.35 | 1.96 | 1.50 | 1.87 | 1.68 | 1.41 | 1.79 |
|  | 1.00 | 0.96 | 0.79 | 2.15 | 2.39 | 2.78 | 2.05 | 1.85 | 3.10 | 2.09 | 2.64 | 2.25 | 1.29 | 1.87 | 1.63 | 1.07 | 1.00 | 1.17 |
|  | 1.00 | 1.22 | 1.18 | 2.88 | 3.64 | 3.14 | 2.59 | 3.34 | 3.95 | 2.43 | 3.25 | 2.21 | 1.73 | 2.64 | 1.99 | 1.94 | 1.43 | 1.25 |
| Mean | 1.05 | | | 3.17 | | | 2.95 | | | 2.46 | | | 1.83 | | | 1.42 | | |
| SEM | 0.07 | | | 0.17 | | | 0.13 | | | 0.13 | | | 0.10 | | | 0.08 | | |

**WB (In Vivo)**


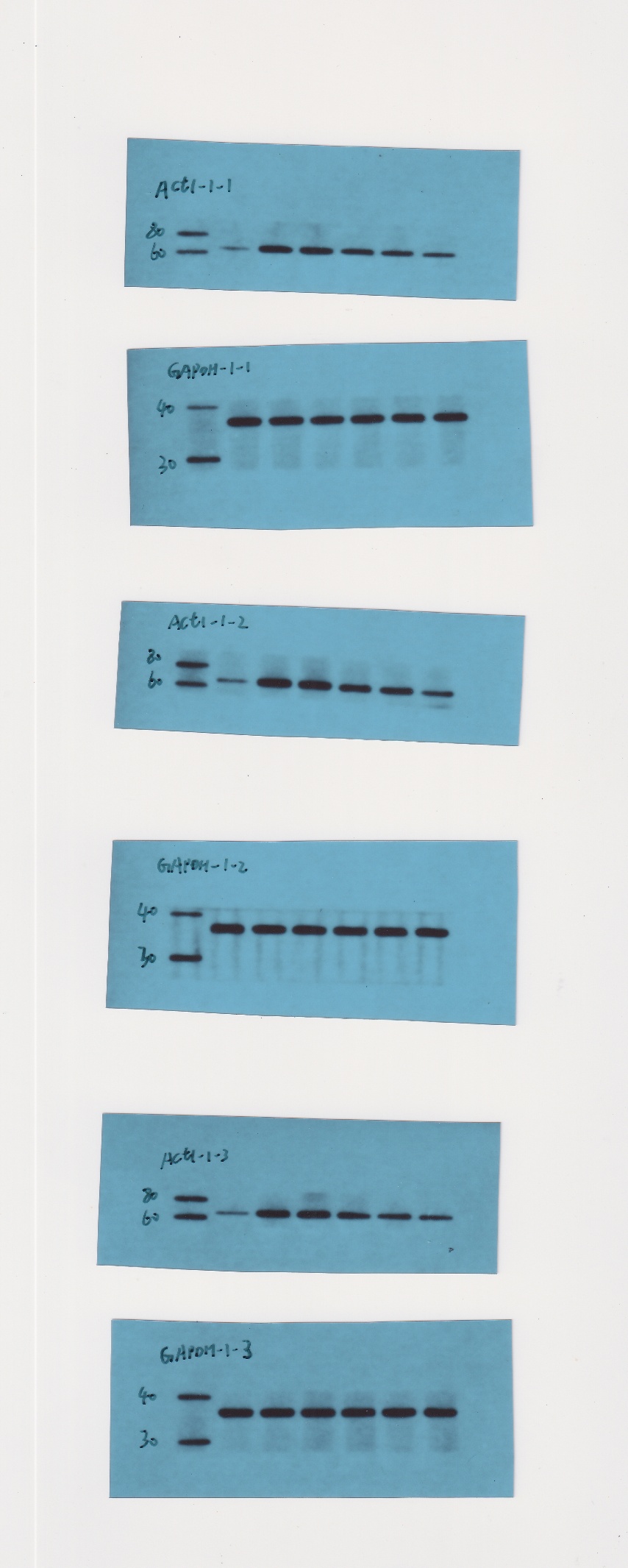

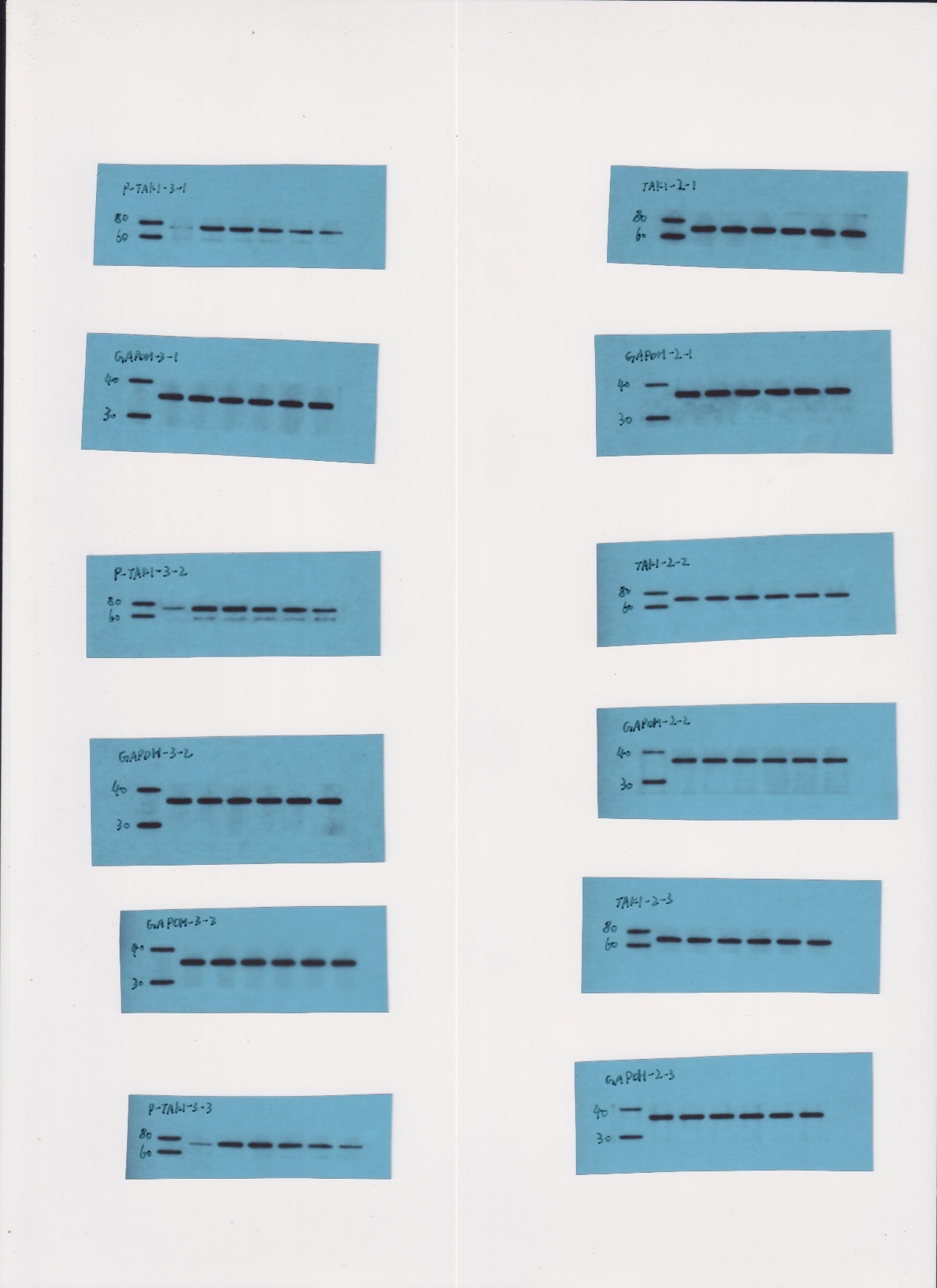

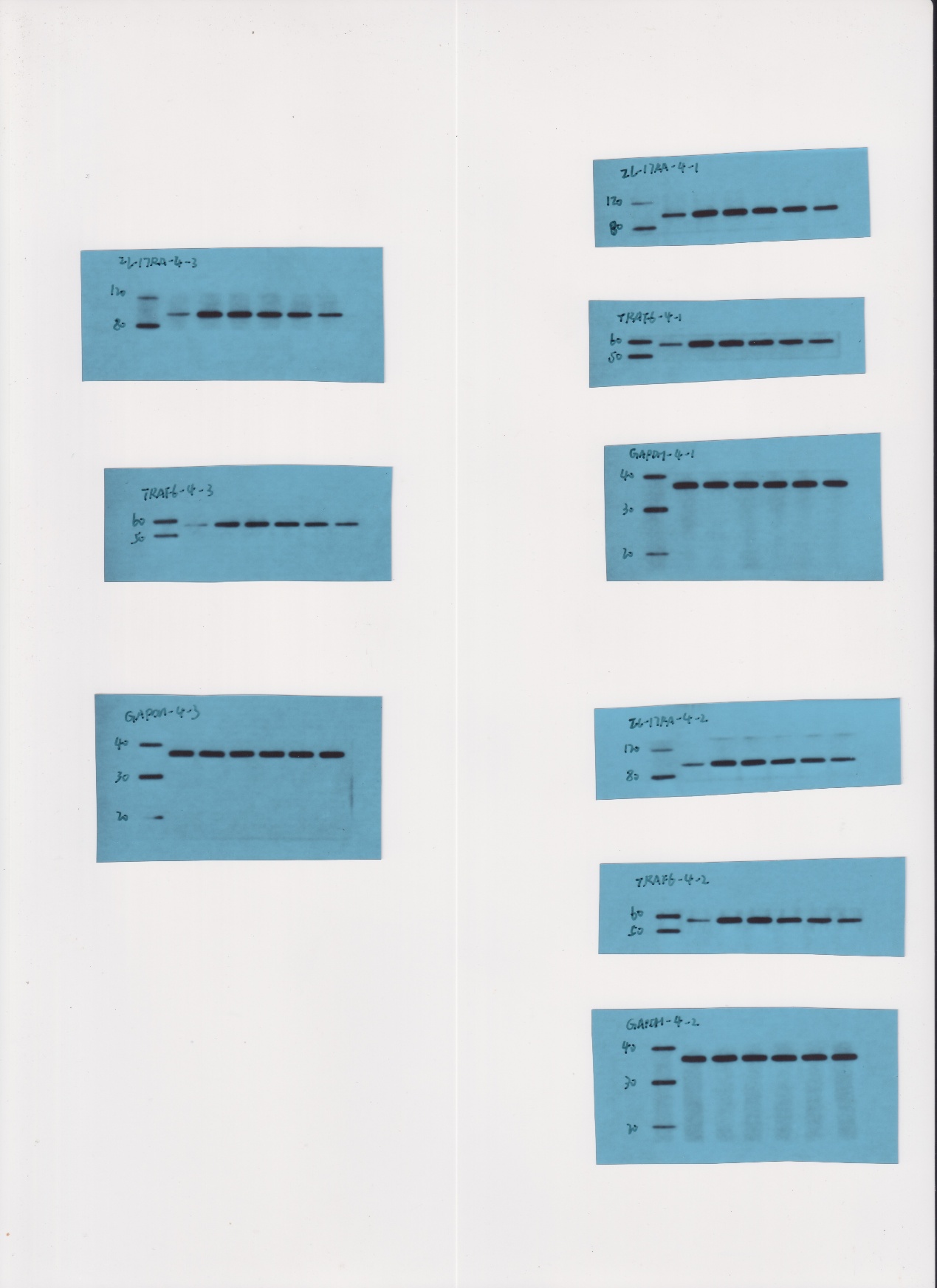

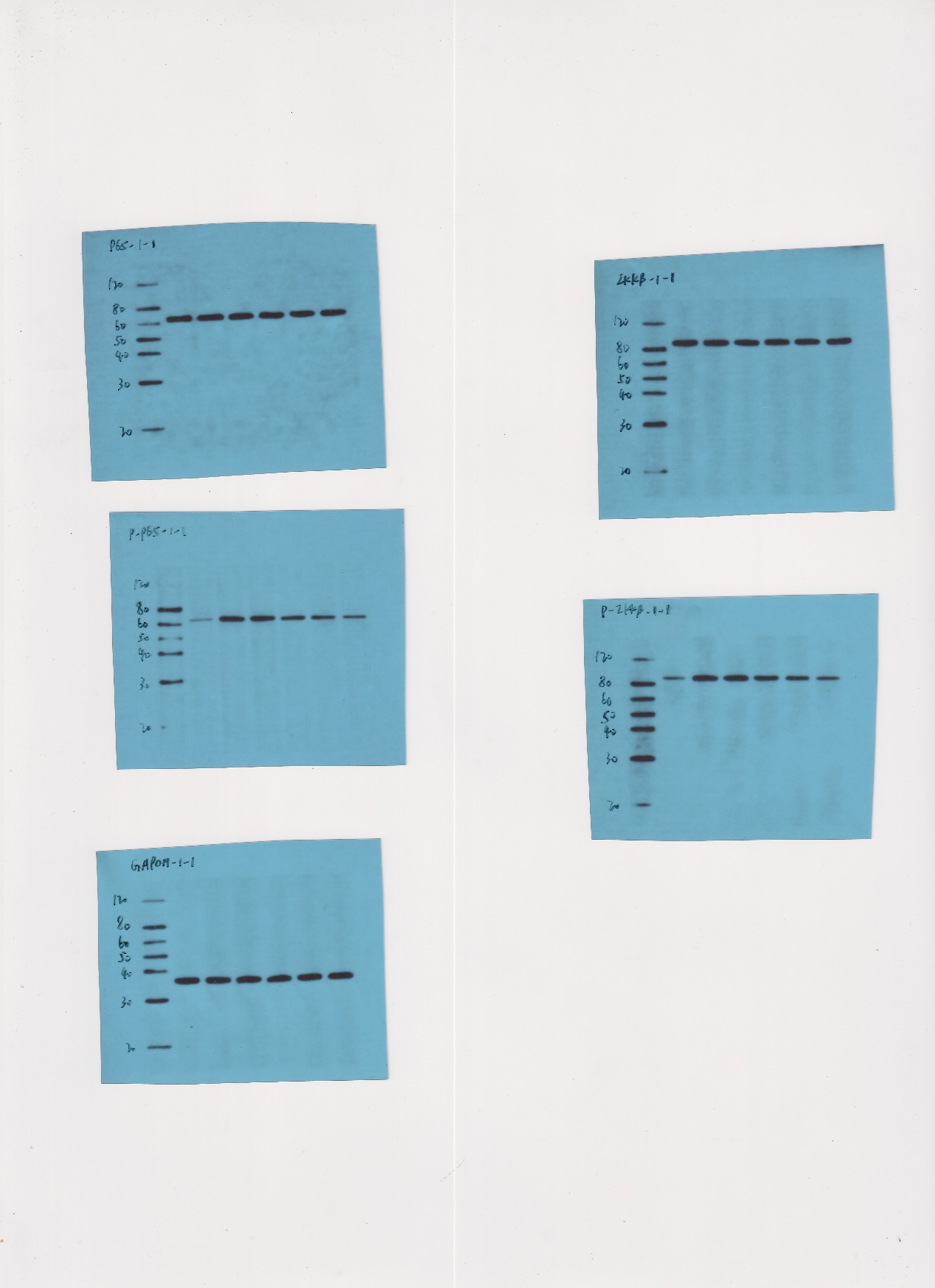

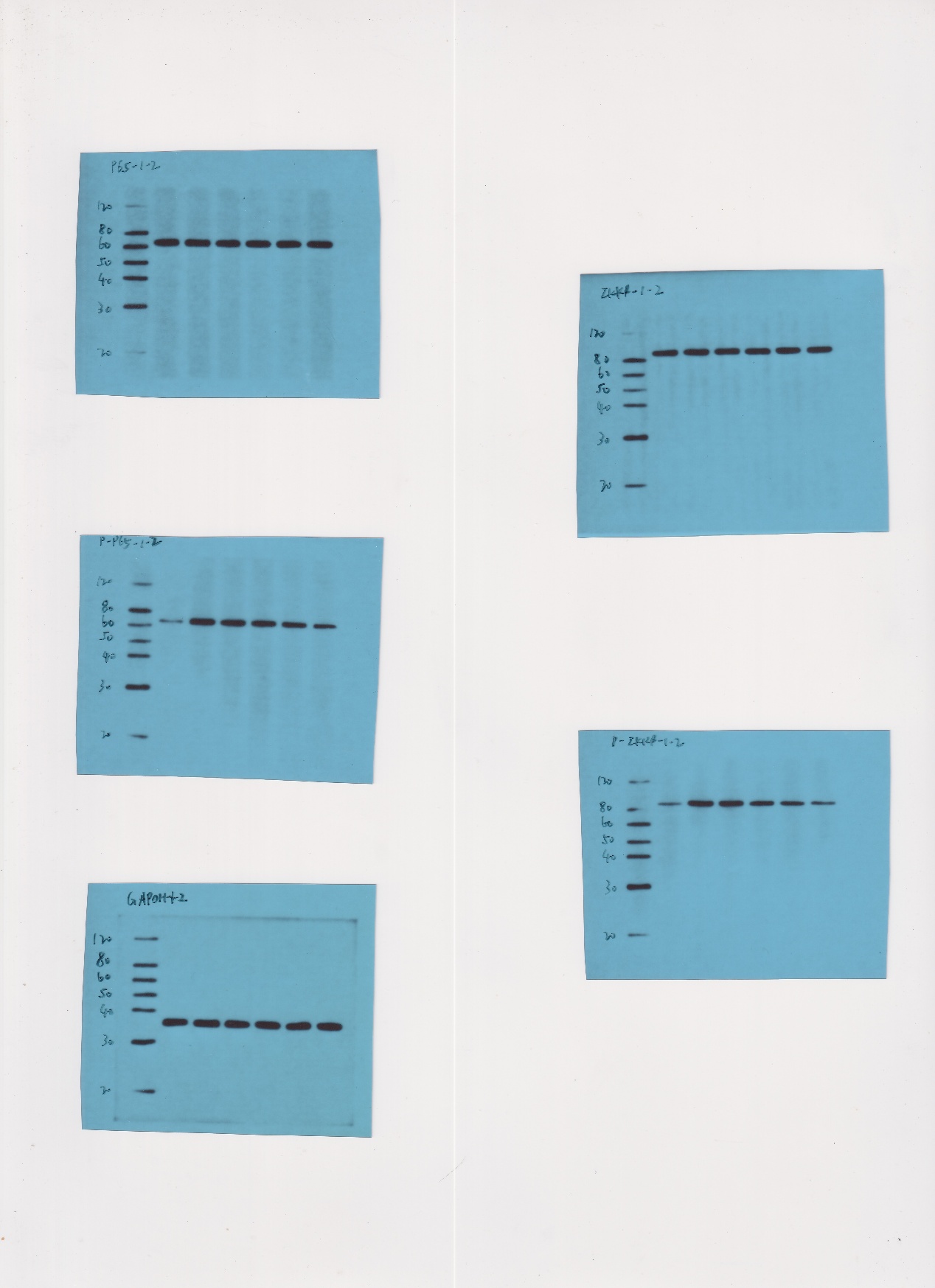

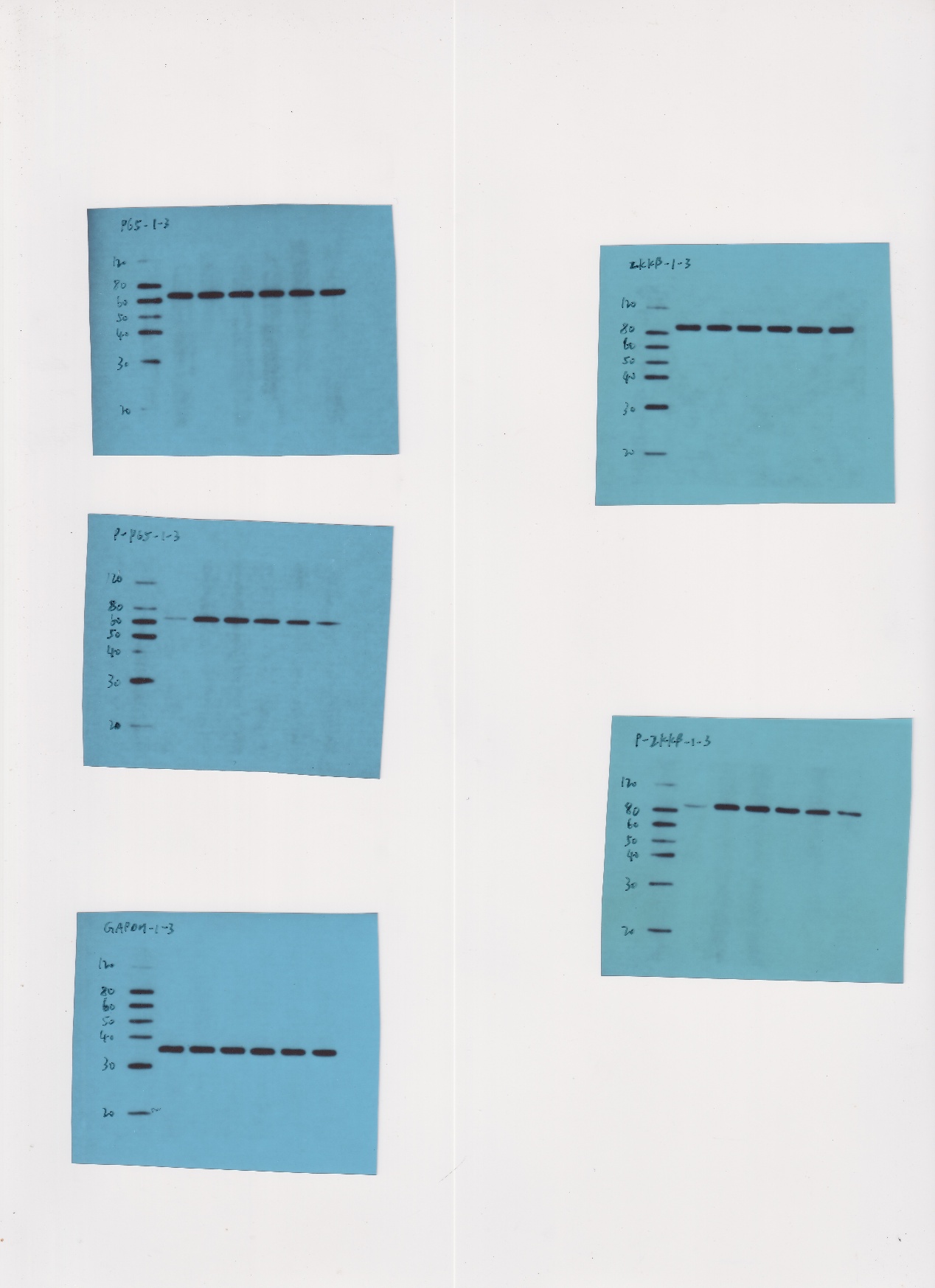

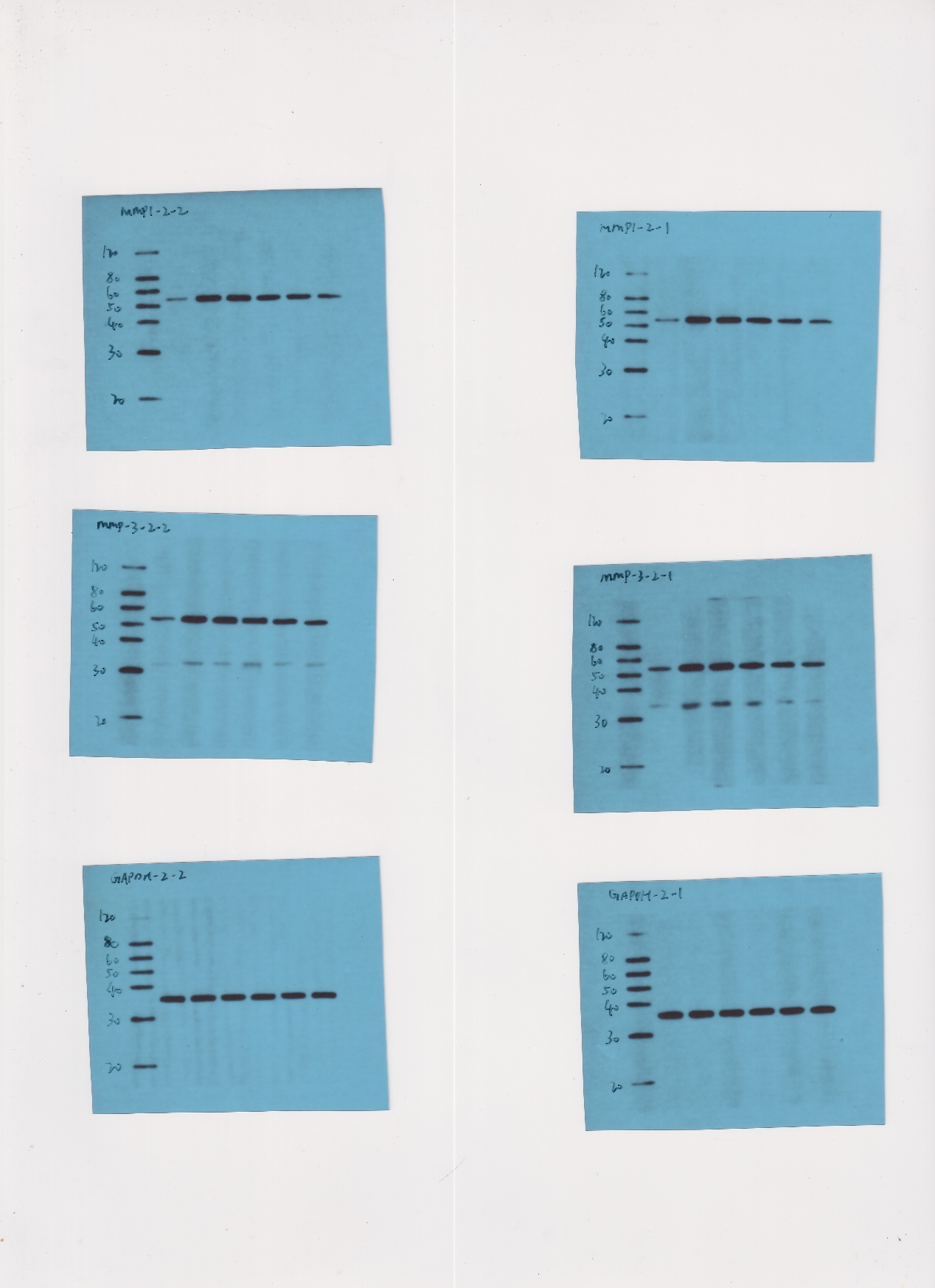

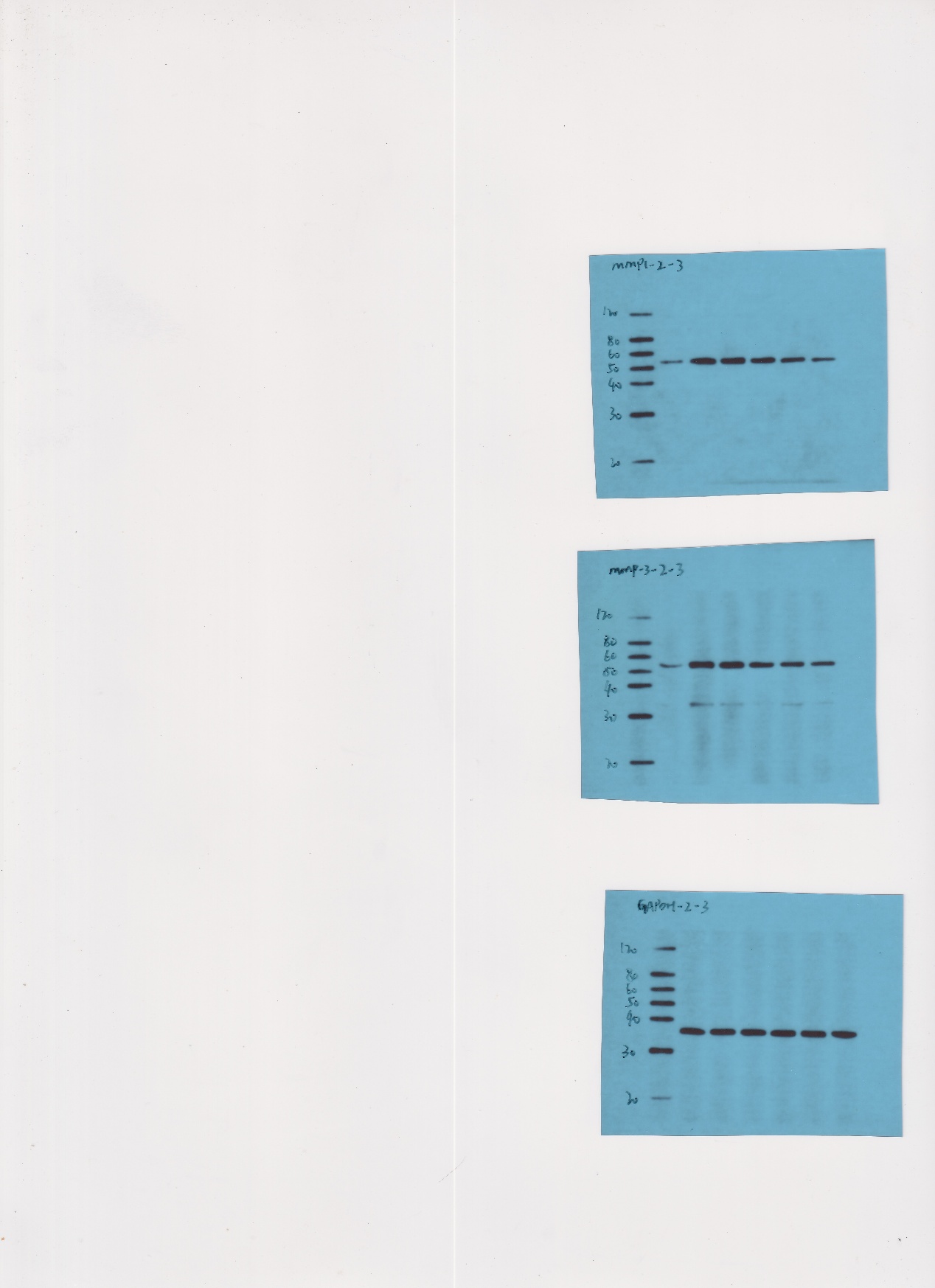


**Part II. HaCaT**

**CCK8 Assays**

**Tabel S11. The CCK8 assay results from the formal experiment**

|  | Control | LL-37(10μM) | LL-37+vicenin-2(2μM) | LL-37+vicenin-2(4μM) | LL-37+vicenin-2(6μM) |
| --- | --- | --- | --- | --- | --- |
| HaCaT Cell Viability (%Control) | 100.00% | 162.54% | 139.59% | 128.45% | 118.14% |
|  | 100.00% | 162.20% | 139.01% | 132.08% | 112.81% |
|  | 100.00% | 168.61% | 140.95% | 130.93% | 120.16% |
| Mean | 100 | 164.5 | 139.9 | 130.5 | 117 |
| SEM | 0 | 2.082 | 0.5749 | 1.071 | 2.192 |

**Tabel S12. CCK8 assay results of LL-37 safety concentration screening**

|  | control | LL-37 (2μM) | LL-37 (4μM) | LL-37 (6μM) | LL-37 (8μM) | LL-37 (10μM) |
| --- | --- | --- | --- | --- | --- | --- |
| HaCaT Cell Viability (%Control) | 100.00% | 108.68% | 121.31% | 136.58% | 121.62% | 145.49% |
|  | 100.00% | 96.81% | 100.00% | 107.26% | 116.56% | 120.50% |
|  | 100.00% | 123.11% | 125.46% | 128.72% | 147.18% | 166.71% |
| Mean | 100 | 109.5 | 115.6 | 124.2 | 128.5 | 144.2 |
| SEM | 0 | 7.604 | 7.887 | 8.762 | 9.477 | 13.35 |

**Tabel S13. CCK8 assay results of Vicenin-2 safety concentration screening**

|  | Control | vicenin-2 2μM | vicenin-2 4μM | vicenin-2 6μM | vicenin-2 8μM | vicenin-2 10μM |
| --- | --- | --- | --- | --- | --- | --- |
| HaCaT Cell Viability (%Control) | 100.00% | 103.68% | 106.21% | 102.43% | 92.47% | 82.42% |
|  | 100.00% | 108.50% | 105.38% | 101.34% | 95.48% | 88.85% |
|  | 100.00% | 105.55% | 102.77% | 104.58% | 99.81% | 95.30% |
| Mean | 100 | 105.9 | 104.8 | 102.8 | 95.92 | 88.86 |
| SEM | 0 | 1.403 | 1.036 | 0.9518 | 2.13 | 3.718 |

**ELISA**

| **Tabel S14.** ELISA Raw Data of TNF-α | | | | | | | |
| --- | --- | --- | --- | --- | --- | --- | --- |
| Sample | OD Value | | | Mean minus blank OD value | Concentration of TNF-α(pg/ml) | AVERAGE | SEM |
| Control | 0.172 | 0.171 | 0.173 | 0.042 | 16.820 | 16.48 | 1.099 |
|  | 0.165 | 0.163 | 0.167 | 0.035 | 14.429 |  |  |
|  | 0.176 | 0.174 | 0.178 | 0.046 | 18.189 |  |  |
| LL-37 | 0.346 | 0.345 | 0.342 | 0.214 | 77.754 | 81.17 | 2.264 |
|  | 0.350 | 0.353 | 0.351 | 0.221 | 80.313 |  |  |
|  | 0.364 | 0.365 | 0.367 | 0.235 | 85.453 |  |  |
| LL-37+vicenin-2(2μM) | 0.328 | 0.327 | 0.330 | 0.198 | 71.927 | 73.47 | 2.636 |
|  | 0.346 | 0.349 | 0.345 | 0.217 | 78.606 |  |  |
|  | 0.321 | 0.325 | 0.322 | 0.193 | 69.872 |  |  |
| LL-37+vicenin-2(4μM) | 0.299 | 0.300 | 0.302 | 0.170 | 61.815 | 62.42 | 3.094 |
|  | 0.318 | 0.316 | 0.319 | 0.188 | 68.062 |  |  |
|  | 0.289 | 0.288 | 0.287 | 0.158 | 57.395 |  |  |
| LL-37+vicenin-2(6μM) | 0.231 | 0.223 | 0.214 | 0.093 | 34.320 | 39.89 | 3.509 |
|  | 0.257 | 0.255 | 0.259 | 0.127 | 46.374 |  |  |
|  | 0.234 | 0.241 | 0.233 | 0.106 | 38.983 |  |  |
|  |  |  |  |  |  |  |  |
|  |  |  |  |  |  |  |  |
| **Tabel S15.** ELISA Raw Data of IL-6 | | | | | | | |
| Sample | OD Value | | | Mean minus blank OD value | Concentration of TNF-α(pg/ml) | AVERAGE | SEM |
| Control | 0.269 | 0.268 | 0.271 | 0.165 | 11.001 | 11.22 | 0.8157 |
|  | 0.297 | 0.295 | 0.294 | 0.191 | 12.724 |  |  |
|  | 0.251 | 0.253 | 0.254 | 0.149 | 9.923 |  |  |
| LL-37 | 0.890 | 0.889 | 0.888 | 0.785 | 69.135 | 74.99 | 3.402 |
|  | 0.932 | 0.933 | 0.935 | 0.829 | 74.924 |  |  |
|  | 0.980 | 0.977 | 0.975 | 0.873 | 80.920 |  |  |
| LL-37+vicenin-2(2μM) | 0.852 | 0.853 | 0.850 | 0.748 | 64.453 | 65.04 | 3.054 |
|  | 0.900 | 0.902 | 0.899 | 0.796 | 70.591 |  |  |
|  | 0.817 | 0.814 | 0.815 | 0.711 | 60.061 |  |  |
| LL-37+vicenin-2(4μM) | 0.702 | 0.706 | 0.705 | 0.600 | 47.615 | 48.4 | 2.42 |
|  | 0.673 | 0.677 | 0.678 | 0.572 | 44.663 |  |  |
|  | 0.751 | 0.754 | 0.755 | 0.649 | 52.933 |  |  |
| LL-37+vicenin-2(6μM) | 0.522 | 0.518 | 0.519 | 0.416 | 29.901 | 26.87 | 1.776 |
|  | 0.485 | 0.487 | 0.483 | 0.381 | 26.956 |  |  |
|  | 0.448 | 0.444 | 0.445 | 0.342 | 23.749 |  |  |
|  |  |  |  |  |  |  |  |
|  |  |  |  |  |  |  |  |
| **Tabel S16.** ELISA Raw Data of IL-8 | | | | | | | |
| Sample | OD Value | | | Mean minus blank OD value | Concentration of TNF-α(pg/ml) | AVERAGE | SEM |
| Control | 0.321 | 0.319 | 0.323 | 0.244 | 39.865 | 39.85 | 1.982 |
|  | 0.353 | 0.355 | 0.352 | 0.276 | 43.271 |  |  |
|  | 0.290 | 0.292 | 0.289 | 0.213 | 36.404 |  |  |
| LL-37 | 1.397 | 1.392 | 1.384 | 1.314 | 139.462 | 139.4 | 6.311 |
|  | 1.308 | 1.307 | 1.306 | 1.230 | 128.448 |  |  |
|  | 1.466 | 1.462 | 1.465 | 1.387 | 150.309 |  |  |
| LL-37+vicenin-2(2μM) | 1.118 | 1.120 | 1.123 | 1.043 | 107.946 | 108.3 | 6.506 |
|  | 1.234 | 1.235 | 1.231 | 1.156 | 119.793 |  |  |
|  | 1.008 | 1.004 | 1.005 | 0.929 | 97.267 |  |  |
| LL-37+vicenin-2(4μM) | 0.948 | 0.954 | 0.951 | 0.874 | 92.533 | 87.49 | 3.661 |
|  | 0.799 | 0.804 | 0.801 | 0.724 | 80.372 |  |  |
|  | 0.913 | 0.918 | 0.916 | 0.839 | 89.571 |  |  |
| LL-37+vicenin-2(6μM) | 0.548 | 0.549 | 0.546 | 0.471 | 60.584 | 59.9 | 4.202 |
|  | 0.624 | 0.627 | 0.628 | 0.549 | 66.806 |  |  |
|  | 0.449 | 0.451 | 0.447 | 0.372 | 52.300 |  |  |
|  |  |  |  |  |  |  |  |
|  |  |  |  |  |  |  |  |
| **Tabel S17.** ELISA Raw Data of IL-1β | | | | | | | |
| Sample | OD Value | | | Mean minus blank OD value | Concentration of TNF-α(pg/ml) | AVERAGE | SEM |
| Control | 0.202 | 0.200 | 0.204 | 0.134 | 9.125 | 9.152 | 0.6525 |
|  | 0.187 | 0.186 | 0.189 | 0.119 | 8.036 |  |  |
|  | 0.218 | 0.220 | 0.217 | 0.150 | 10.296 |  |  |
| LL-37 | 0.647 | 0.645 | 0.646 | 0.578 | 33.468 | 33.86 | 1.585 |
|  | 0.716 | 0.712 | 0.715 | 0.646 | 36.780 |  |  |
|  | 0.601 | 0.600 | 0.604 | 0.534 | 31.332 |  |  |
| LL-37+vicenin-2(2μM) | 0.500 | 0.501 | 0.503 | 0.433 | 26.447 | 26.68 | 1.323 |
|  | 0.461 | 0.465 | 0.462 | 0.395 | 24.511 |  |  |
|  | 0.554 | 0.555 | 0.556 | 0.487 | 29.076 |  |  |
| LL-37+vicenin-2(4μM) | 0.404 | 0.401 | 0.412 | 0.338 | 21.562 | 21.17 | 1.066 |
|  | 0.431 | 0.427 | 0.429 | 0.361 | 22.786 |  |  |
|  | 0.365 | 0.357 | 0.362 | 0.293 | 19.158 |  |  |
| LL-37+vicenin-2(6μM) | 0.295 | 0.291 | 0.287 | 0.223 | 15.060 | 16.97 | 1.052 |
|  | 0.354 | 0.353 | 0.352 | 0.285 | 18.692 |  |  |
|  | 0.326 | 0.330 | 0.322 | 0.258 | 17.150 |  |  |

**Tabel S18. Raw data of RT-qPCR for IL-16, TNF-α and IL-1β in vitro**

|  | **Control** | **LL-37** | **LL-37+vicenin-2(2μM)** | **LL-37+vicenin-2(4μM)** | **LL-37+vicenin-2(6μM)** |
| --- | --- | --- | --- | --- | --- |
| **Homo GAPDH** | 15.566 | 15.656 | 16.127 | 16.018 | 16.347 |
|  | 15.464 | 15.762 | 15.889 | 15.844 | 16.294 |
|  | 15.504 | 15.919 | 15.731 | 15.943 | 16.151 |

|  | **Control** | **LL-37** | **LL-37+vicenin-2(2μM)** | **LL-37+vicenin-2(4μM)** | **LL-37+vicenin-2(6μM)** |
| --- | --- | --- | --- | --- | --- |
| **Homo IL-6** | 28.099 | 26.877 | 27.532 | 27.589 | 28.505 |
|  | 28.139 | 27.136 | 27.220 | 27.750 | 28.327 |
|  | 27.986 | 27.016 | 27.332 | 27.628 | 28.170 |
|  |  |  |  |  |  |
| Δt | 12.533 | 11.221 | 11.405 | 11.571 | 12.158 |
|  | 12.675 | 11.373 | 11.331 | 11.906 | 12.033 |
|  | 12.482 | 11.097 | 11.601 | 11.685 | 12.019 |
|  |  |  |  |  |  |
| ΔΔt | 0.000 | -1.312 | -1.128 | -0.962 | -0.375 |
|  | 0.000 | -1.302 | -1.344 | -0.769 | -0.642 |
|  | 0.000 | -1.385 | -0.881 | -0.797 | -0.463 |
|  |  |  |  |  |  |
| 2-ΔΔt | 1.000 | 2.482 | 2.185 | 1.948 | 1.297 |
|  | 1.000 | 2.465 | 2.539 | 1.704 | 1.560 |
|  | 1.000 | 2.612 | 1.842 | 1.737 | 1.378 |
| Mean | 1.008 | 2.512 | 2.208 | 1.812 | 1.393 |
| SEM | 0.062 | 0.097 | 0.124 | 0.069 | 0.099 |

|  | **Control** | **LL-37** | **LL-37+vicenin-2(2μM)** | **LL-37+vicenin-2(4μM)** | **LL-37+vicenin-2(6μM)** |
| --- | --- | --- | --- | --- | --- |
| **Homo TNF-α** | 32.378 | 30.208 | 30.909 | 31.239 | 31.990 |
|  | 32.457 | 30.178 | 30.781 | 31.326 | 32.167 |
|  | 32.825 | 30.324 | 30.714 | 31.158 | 31.798 |
|  |  |  |  |  |  |
| Δt | 16.812 | 14.552 | 14.782 | 15.221 | 15.643 |
|  | 16.993 | 14.416 | 14.892 | 15.482 | 15.873 |
|  | 17.321 | 14.405 | 14.983 | 15.215 | 15.647 |
|  |  |  |  |  |  |
| ΔΔt | 0.000 | -2.260 | -2.030 | -1.591 | -1.169 |
|  | 0.000 | -2.577 | -2.101 | -1.511 | -1.120 |
|  | 0.000 | -2.916 | -2.338 | -2.106 | -1.674 |
|  |  |  |  |  |  |
| 2-ΔΔt | 1.000 | 4.790 | 4.085 | 3.013 | 2.248 |
|  | 1.000 | 5.969 | 4.290 | 2.850 | 2.173 |
|  | 1.000 | 7.550 | 5.057 | 4.305 | 3.191 |
| Mean | 1.008 | 2.512 | 2.208 | 1.812 | 1.393 |
| SEM | 0.062 | 0.097 | 0.124 | 0.069 | 0.099 |

|  | **Control** | **LL-37** | **LL-37+vicenin-2(2μM)** | **LL-37+vicenin-2(4μM)** | **LL-37+vicenin-2(6μM)** |
| --- | --- | --- | --- | --- | --- |
| **Homo TNF-α** | 25.463 | 23.682 | 23.992 | 24.301 | 25.374 |
|  | 25.404 | 23.423 | 24.269 | 24.358 | 25.045 |
|  | 25.328 | 23.830 | 24.164 | 24.542 | 25.184 |
|  |  |  |  |  |  |
| Δt | 9.897 | 8.026 | 7.865 | 8.283 | 9.027 |
|  | 9.940 | 7.661 | 8.380 | 8.514 | 8.751 |
|  | 9.824 | 7.911 | 8.433 | 8.599 | 9.033 |
|  |  |  |  |  |  |
| ΔΔt | 0.000 | -1.871 | -2.032 | -1.614 | -0.870 |
|  | 0.000 | -2.279 | -1.560 | -1.426 | -1.189 |
|  | 0.000 | -1.913 | -1.391 | -1.225 | -0.791 |
|  |  |  |  |  |  |
| 2-ΔΔt | 1.000 | 3.658 | 4.089 | 3.062 | 1.828 |
|  | 1.000 | 4.855 | 2.949 | 2.687 | 2.279 |
|  | 1.000 | 3.767 | 2.623 | 2.337 | 1.730 |
| Mean | 0.994 | 4.083 | 3.208 | 2.707 | 1.904 |
| SEM | 0.062 | 0.081 | 0.123 | 0.138 | 0.116 |

**WB (in vitro)**


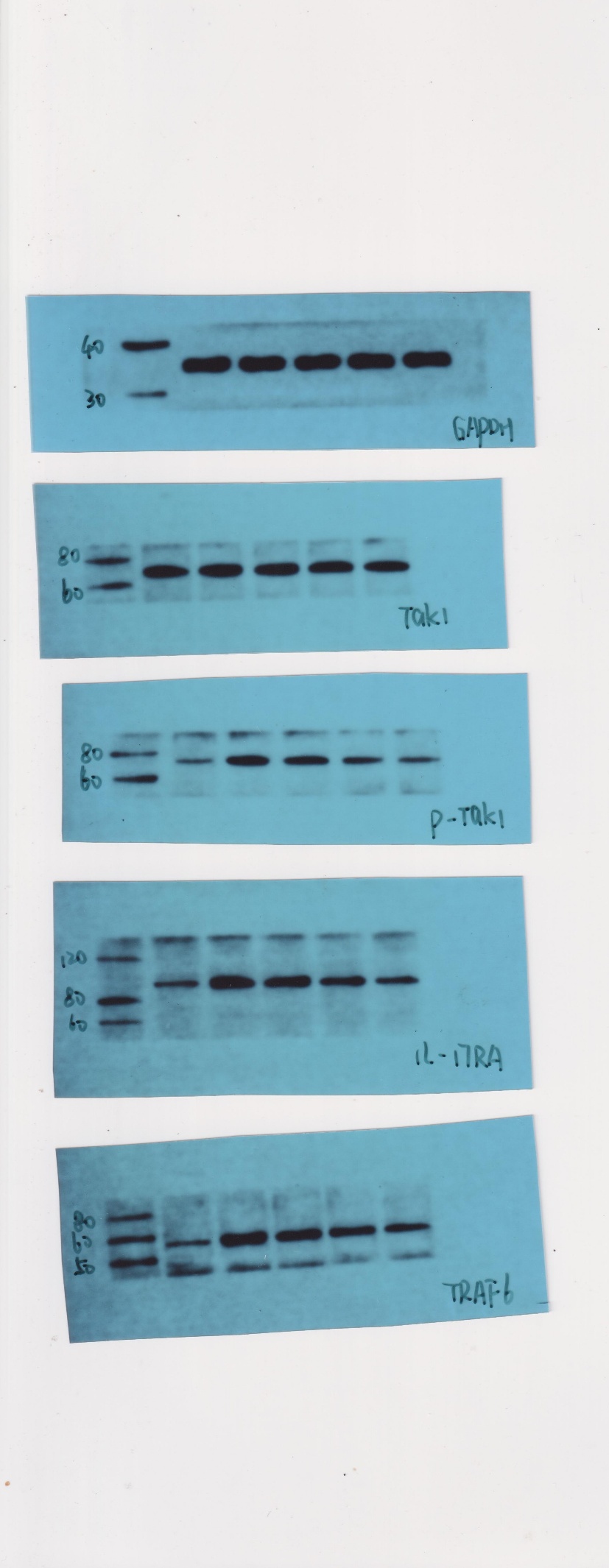


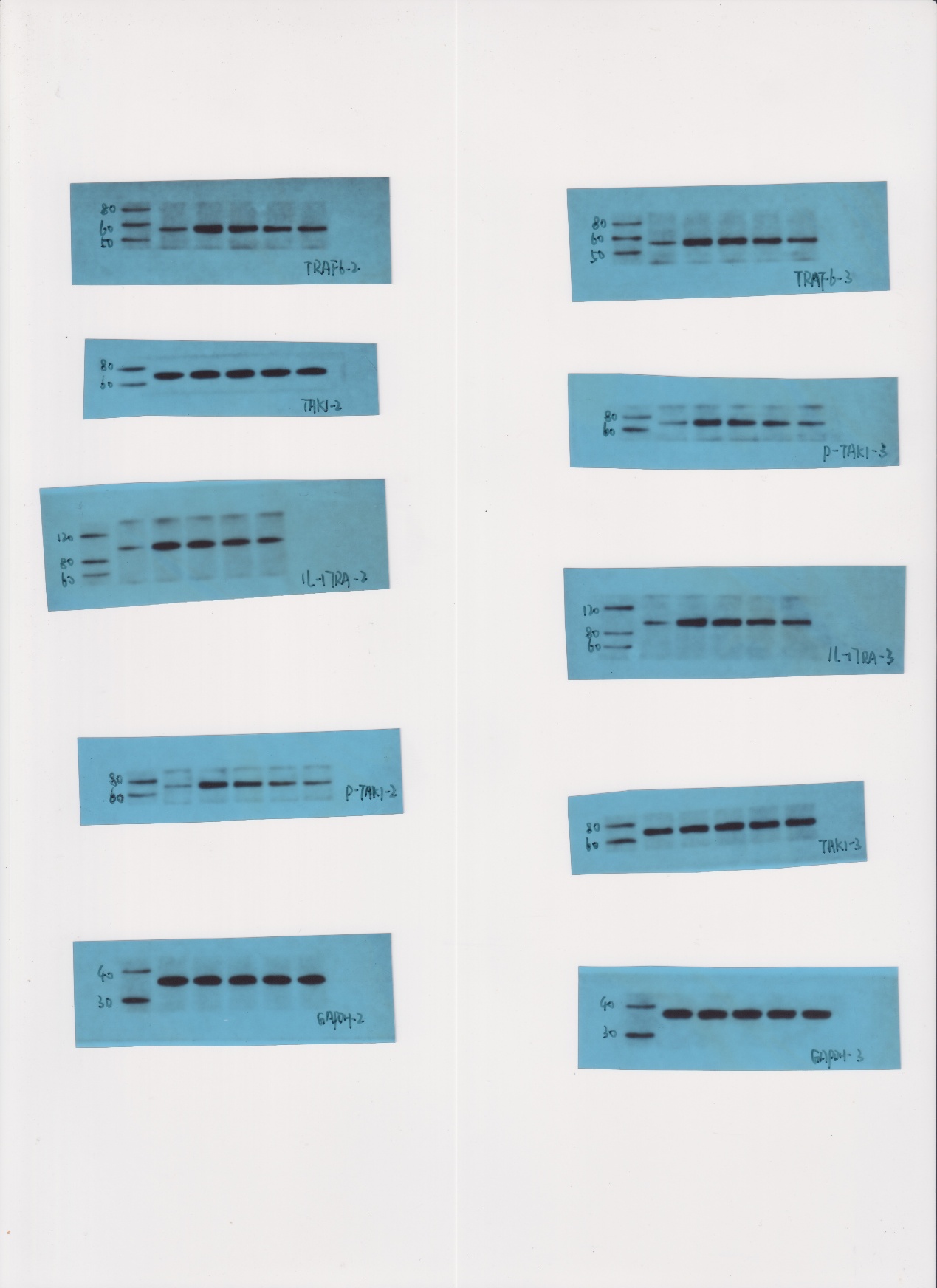


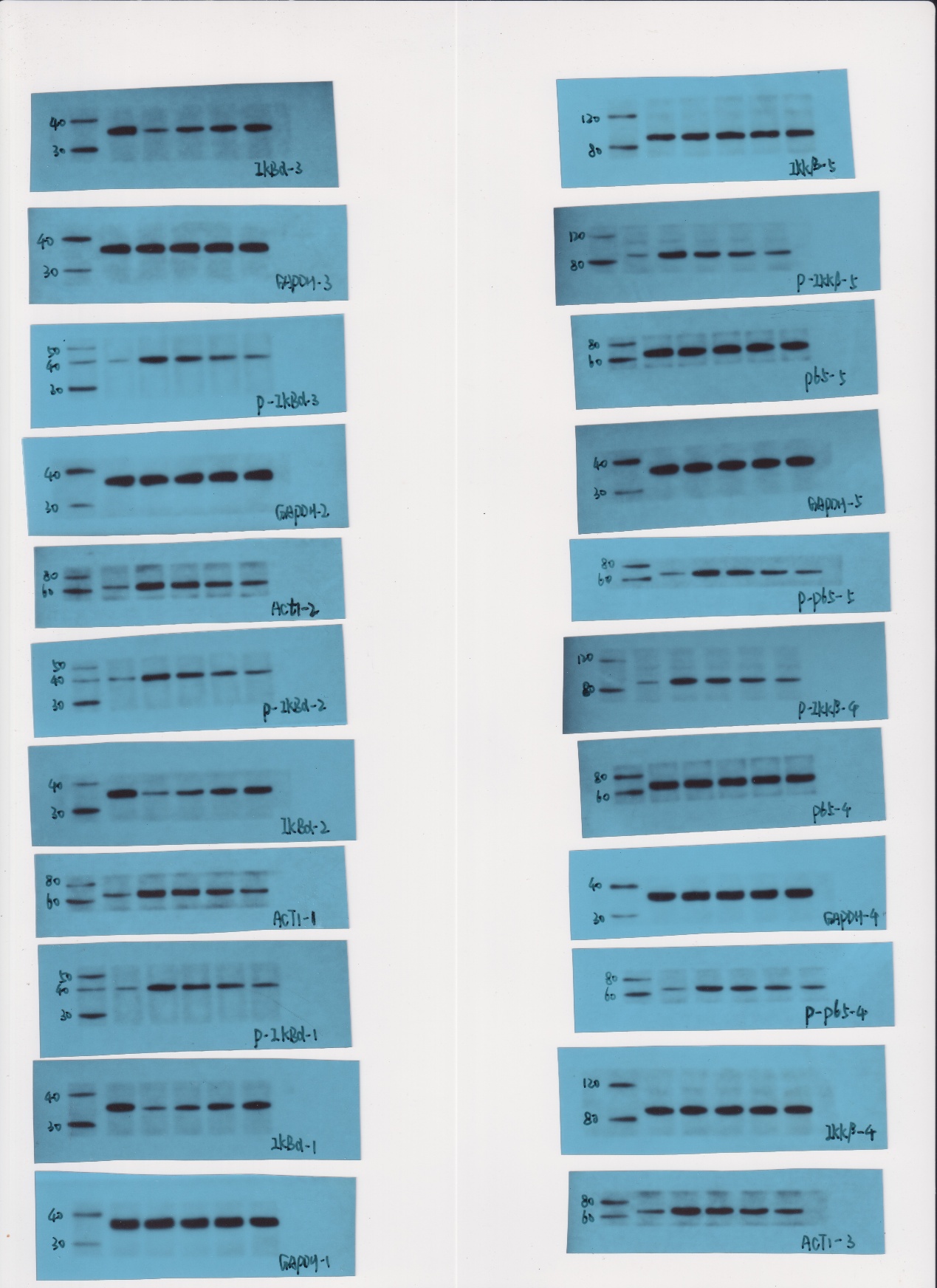


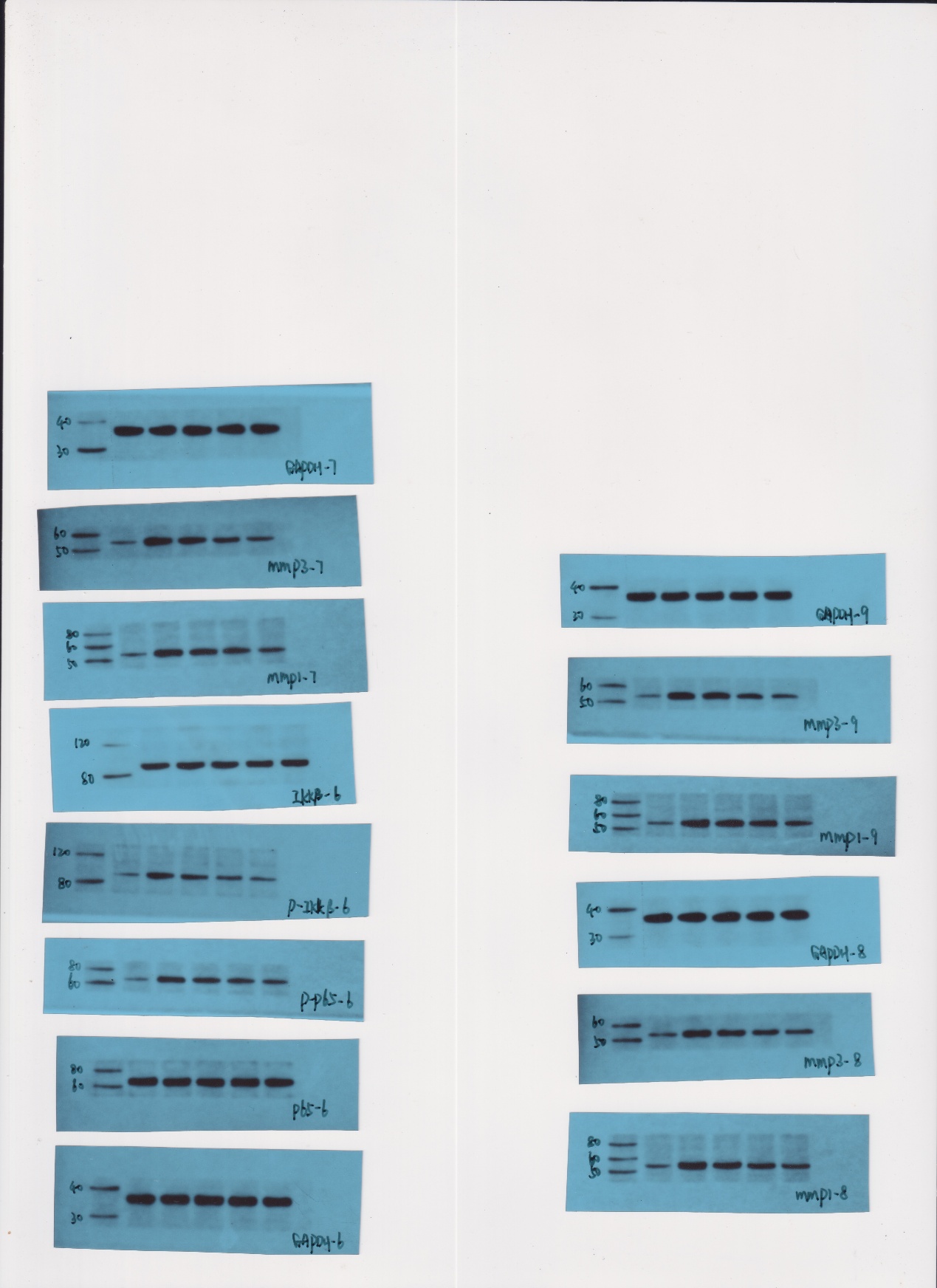

Supplement: Supplementary file 1 [file Supplementaryfile1.docx]
